# Impact of the Food Additive Titanium Dioxide (E171) on Gut Microbiota-Host Interaction

**DOI:** 10.3389/fnut.2019.00057

**Published:** 2019-05-14

**Authors:** Gabriela Pinget, Jian Tan, Bartlomiej Janac, Nadeem O. Kaakoush, Alexandra Sophie Angelatos, John O'Sullivan, Yen Chin Koay, Frederic Sierro, Joel Davis, Shiva Kamini Divakarla, Dipesh Khanal, Robert J. Moore, Dragana Stanley, Wojciech Chrzanowski, Laurence Macia

**Affiliations:** ^1^The Charles Perkins Centre, The University of Sydney, Sydney, NSW, Australia; ^2^Faculty of Medicine and Health, School of Medical Sciences, The University of Sydney, Sydney, NSW, Australia; ^3^Sydney Nano Institute, The University of Sydney, Sydney, NSW, Australia; ^4^Human Health, Nuclear Science & Technology and Landmark Infrastructure (NSTLI), Australian Nuclear Science and Technology Organisation, Sydney, NSW, Australia; ^5^School of Medical Sciences, University of New South Wales, Sydney, NSW, Australia; ^6^Department of Cardiology, Charles Perkins Centre, Royal Prince Alfred Hospital, Heart Research Institute, University of Sydney, Sydney, NSW, Australia; ^7^Sydney Pharmacy School, The University of Sydney, Sydney, NSW, Australia; ^8^School of Science, RMIT University, Bundoora, VIC, Australia; ^9^School of Health, Medical and Applied Sciences, Central Queensland University, Rockhampton, QLD, Australia

**Keywords:** biofilm, gut microbiota, immune cells, inflammation, titanium dioxide

## Abstract

The interaction between gut microbiota and host plays a central role in health. Dysbiosis, detrimental changes in gut microbiota and inflammation have been reported in non-communicable diseases. While diet has a profound impact on gut microbiota composition and function, the role of food additives such as titanium dioxide (TiO_2_), prevalent in processed food, is less established. In this project, we investigated the impact of food grade TiO_2_ on gut microbiota of mice when orally administered via drinking water. While TiO_2_ had minimal impact on the composition of the microbiota in the small intestine and colon, we found that TiO_2_ treatment could alter the release of bacterial metabolites *in vivo* and affect the spatial distribution of commensal bacteria *in vitro* by promoting biofilm formation. We also found reduced expression of the colonic mucin 2 gene, a key component of the intestinal mucus layer, and increased expression of the beta defensin gene, indicating that TiO_2_ significantly impacts gut homeostasis. These changes were associated with colonic inflammation, as shown by decreased crypt length, infiltration of CD8^+^ T cells, increased macrophages as well as increased expression of inflammatory cytokines. These findings collectively show that TiO_2_ is not inert, but rather impairs gut homeostasis which may in turn prime the host for disease development.

## Introduction

Bacterial species that inhabit the colon interact with the host, promoting the development and function of immune cells locally and systemically. These interactions are mediated by bacterially derived metabolites such as short-chain fatty acids (SCFAs), which have been identified as critical inducers of immune subsets (1–3) key for protecting mice from disease development (2–5), emphasizing the role of the microbiota in gut homeostasis and host health.

The colonic epithelium acts as a physical barrier between the host and the gut microbiota. The secretion of mucus by goblet cells provides a barrier to microbial infiltration. Further, Paneth cells release antimicrobial peptides that protect against pathogen invasion as well as regulate gut microbiota composition (6). Expression of tight junction proteins by enterocytes also limits bacterial penetration. Epithelial function can be regulated by the gut microbiota via SCFAs, by stimulating mucus production (7) and tight junction assembly (8). In contrast, dysbiosis, marked by detrimental changes in gut microbiota composition, triggers increased gut permeability and gut inflammation (9). Alterations in antimicrobial peptide production, mucus layer thickness and/or epithelial permeability have been implicated in the development of a broad range of diseases such as colitis and colorectal cancer (10). These diseases have also been linked to abnormal interactions between the host epithelium and the gut microbiota through the formation of biofilm. Biofilms consist of aggregates of adherent and planktonic bacteria protected by an extracellular matrix and have been observed in the proximal colon of patients diagnosed with such diseases (11). The mechanisms behind the formation and the role of biofilm in the gut are not fully understood, but biofilm formation has been shown to impact both disease development and resolution. Both in a colitis rat model and in humans, biofilm in the colon has been shown to facilitate pathobiont adherence to the epithelium and translocation to the host (12, 13). In human inflammatory bowel disease, biofilm formation at the site of epithelial wound healing has been shown to negatively affect healing by impairing epithelialization and tissue repair (14). Finally, a recent study has shown that inoculation of germ-free mice with biofilm positive human colon inocula was carcinogenic (15).

The identification of environmental factors that can affect gut homeostasis is thus a critical first step in preventing the development of so-called “western lifestyle diseases,” encompassing autoimmune, allergic and metabolic diseases. A broad range of environmental factors can affect gut homeostasis, with diet composition being the major driver (16). Western-like diets enriched in fat and simple carbohydrates and deficient in dietary fiber have been shown to trigger dysbiosis, increases gut permeability and inflammation (16). While the impact of these macronutrients on gut homeostasis has been extensively studied (17), the role of food additives prevalent in processed food remains poorly defined. Food additives are used to improve the texture, preservation and aesthetics of food. Food grade titanium dioxide (TiO_2_) or E171, is a whitening agent present in over 900 commonly consumed food products. The average adult consumes between 0.7 and 5.9 mg of TiO_2_ per kg of body weight (BW) per day throughout their life and children are the most exposed, consuming up to 32.4 mg TiO_2_/kg BW/day in maximally exposed individuals (18). Despite the fact that regulatory bodies do not define strict guidelines around its consumption, new evidence from animal studies has emerged, highlighting that TiO_2_ may potentiate cancer development (19) and exacerbate inflammatory bowel disease (20).

The effect of TiO_2_ on gut homeostasis is poorly understood yet evidence suggests that TiO_2_ interacts with gut epithelial cells. *In vivo* and *in vitro* studies have demonstrated the accumulation of TiO_2_ in the mucus layer (21) and its uptake by colonic epithelial cells (22, 23). A study in rats has shown that TiO_2_ affects immune cells in the Peyer's patches associated with a decreased regulatory T cell proportion (19). However, the impact of TiO_2_ on colonic immune cells, the site where microbiota is the densest, has never been investigated. While the impact of TiO_2_ on the colonic microbiota has been previously investigated in a short term study (2.5 mg TiO_2_/kg BW/day for 1 week) (24) and using a high dose (100 mg TiO_2_/kg BW/day) for up to 4 weeks (25), the impact of TiO_2_ on the small intestine microbiota is unknown.

The aim of the present study is to establish the effects of food grade TiO_2_ on gut homeostasis *in vivo*. We investigated the impact of physiological doses (2 and 10 mg TiO_2_/kg BW/day) and a high dose of TiO_2_ (50 mg TiO_2_/kg BW/day) on mouse colonic and small intestine microbiota composition and function, epithelial function and mucosal inflammation after 3–4 weeks of treatment via drinking water.

## Materials and Methods

### E171 Characterization

#### Size and Morphology

Food grade TiO_2_ was purchased from All Color Supplies PTY. Average hydrodynamic diameter, polydispersity index and zeta potential of the TiO_2_ nanoparticles dispersed in drinking water were determined with a Malvern Zetasizer Nano ZS at 25°C. The dispersion was measured 3 times for both size and zeta potential. The size distribution and shape of the TiO_2_ nanoparticles dispersed in mice drinking water were determined using a NanoSight NS300 (equipped with a sCMOS camera) at 25°C. The dispersion was measured 5 times (1 min per measurement). The size distribution and shape of the TiO_2_ nanoparticles dispersed in drinking water were further investigated using a Zeiss Ultra Plus scanning electron microscope operated at an accelerating voltage of 10 kV. A drop of the nanoparticle dispersion was allowed to dry on a stub, after which ~20 Å of platinum metal was sputter coated onto the stub under vacuum to prevent charging.

### Crystal Structure and Elemental Composition

A D8 Advance Bruker diffractometer was used to conduct the X-ray powder diffraction (XRD) analysis in a flat plate geometry using Ni-filtered Cu Kα radiation and a Bruker Lynx eye detector. The XRD patterns were acquired from 10 to 100° 2θ with a step size of 0.02° and a count time of 0.1 s. Elemental composition was determined using X-ray photoelectron spectroscopy (XPS) with an Al Kα monochromator X-ray source. A survey scan was acquired at 100 eV pass energy between 0 and 1,400 eV. High resolution spectra for individual elements were collected at 100 Ca + 0.05 Ga. Elemental composition was calculated from the high-resolution spectra using CasaXPS with measurements done in triplicate.

### Mice and TiO_2_ Dosage Information

Five to six week-old male C57BL/6JAusb mice from Australian Bio Resources were maintained under specific-pathogen-free conditions. All experimental procedures involving animals were approved by the University of Sydney Animal Ethics Committee under protocol number 2014/696. Mice were cohoused with water and food (AIN93G; Specialty Feeds) access *ad libitum*. Titanium dioxide (E171) was added to water and sonicated daily. TiO_2_ was administered in drinking water at doses of 0, 2, 10, and 50 mg TiO_2_/kg BW/day, which was calculated based on the water intake measured per cage. At week 4, mice were euthanized using CO_2_ asphyxiation.

### Colonic Immune Cell Isolation and Flow Cytometry

Pieces of colon were incubated at 37°C for 40 min in Hank's Balanced Salt Solution (HBSS; Gibco) with 5 mM EDTA, 5% FBS (Gibco) and 15 mM HEPES (Gibco). Intraepithelial lymphocytes were discarded and the remaining tissue was incubated at 37°C for 1 h in HBSS (Gibco) with 6.7 mg/ml collagenase type IV (Gibco), 10% FBS (Gibco) and 15 mM HEPES (Gibco). Cells were passed through a 70 μm mesh and lymphocytes enriched via percoll gradient of 80% and 40% (GE Life Sciences). The list of antibodies used for flow cytometry is in the Supplementary Methods. Viability was determined using the LIVE/DEAD Fixable Blue Dead Cell stain kit (Invitrogen). Flow cytometry was performed on a LSRII flow cytometer (BD Biosciences) and data analysis with FlowJo software (Treestar Inc., Ashland, OR, USA).

### RNA Extraction and Quantitative Real-Time PCR

Total tissue RNA was extracted using TRI Reagent (Sigma) and converted into cDNA using iScript RT Supermix (BioRad) according to both manufacturer's instructions. qPCR was performed on a LightCycler 480 (Roche) using SYBR Green (Biorad) with primer sequences listed in Supplementary Table 1.

### Acetate and Trimethylamine (TMA) Quantification

Quantitative measurements of acetate and TMA in plasma were determined by nuclear magnetic resonance spectroscopy (NMR). Briefly, plasma was filtered through a 3 kDa membrane filter (Merck Millipore) and polar metabolites extracted from the aqueous phase of a water:chloroform:methanol mixture. Samples, containing 4,4-dimethyl-4-silapentane-1-sulfonic acid as an internal standard, were analyzed on a Bruker 600 MHz NMR.

### Plasma Metabolomic Screening

A hydrophilic interaction chromatography LC-MS/MS method was used for choline detection in plasma as described previously (26). The LC was connected to an AB Sciex Triple Quad 5,500 mass spectrometer run in positive ion mode. Data analysis was done on software Multi-Quant 3.0 for MRM Q1/Q3 peak integration.

### Nanolive Imaging

*Escherichia coli* K-12 MG1655 (*E. coli*) or *Enterococcus faecalis* NCTC 775 (*E. faecalis*) were incubated for 7 h at 37°C, 5% CO_2_ with Luria-Bertani (LB) broth containing E171 at indicated concentrations and then fixed in 3% formalin overnight. Cells were resuspended in PBS and visualized using a Nanolive 3D cell explorer. False colors were applied to images based on refractive index using STEVE software.

### Biofilm Visualization

#### Biofilm Formation Assay *in vitro* on Cultured *E. coli* and *E. faecalis*

The *in vitro* biofilm formation assay was based on a previously published protocol (13). Overnight culture in quadruplicates of *E. coli* (low salt LB broth; Beckton Dickinson), *E. faecalis* (tryptone soya broth supplemented with 0.25% glucose; Sigma Aldrich) or *Staphylococcus epidermidis* NCTC 6512 (LB broth) was adjusted to OD of 0.5 at 600 nm and 100 μl of each bacterial culture was plated on separate round bottom 96-well tissue culture plates. A further 100 μl of appropriate media supplemented with TiO_2_ was added to achieve the indicated final concentrations. TiO_2_ at the different final concentrations in media alone was used as background controls. Plates were incubated at 37°C aerobically on a shaker (Ratek, 70 rpm) for either 24, 48, or 72 h.

#### Biofilm Formation Assay From Colonic Commensal Bacteria

Two hundred microliters of colon homogenates were cultured in quadruplicates in flat bottom 96-well-plates containing supplemented tryptic soy broth [sTSY: 30 g/L tryptic soy broth (Oxoid) with 5 g/L yeast extract, 5% L-cysteine, 50 mg/L hemin and 1 mg/L medanione (all from Sigma-Aldrich) to yield 0.05 mg/μl (w/v)] for 24 h, aerobically at 37°C at 70 rpm. Samples were diluted 1:100 in fresh sTSY containing TiO_2_ at indicated doses and incubated for 5 days. After planktonic cell removal, biofilm was stained with crystal violet (CV). Briefly, plates were washed 3 times with water, air dried and stained with 1% CV (Sigma-Aldrich) for 30 min. After 4 washes in water and air drying, 95% ethanol was added for 15 min. Absorbance was recorded at 595 nm on a microplate reader (Tecan Infinite M1000).

#### Resazurin Viability Assay

Biofilm formation was also quantified based on Resazurin viability assay as previously described (27). Briefly, culture media was removed and wells washed once with phosphate-buffered saline (PBS). Then, media with 10% Resazurin (Sigma-Aldrich) was added to each well. The plates were incubated in the dark at 37°C and fluorescence intensity measured every 15 min (excitation 570 nm, emission 585 nm). TiO_2_ only controls were used to subtract background.

### Bacteria 16S rRNA Gene Amplicon Sequencing and Bioinformatics

DNA from fecal samples or entire contents of small intestine lumen were extracted by mechanical disruption using a Fastprep (MP Biomedicals) using autoclaved glass beads (G8772 and G1145; Sigma-Aldrich) in lysis buffer [500 mM NaCl, 50 mM Tris-HCl (pH 8), 50 mM EDTA, 4% SDS] followed by 15 min incubation at 95°C. DNA was precipitated in 10M ammonium acetate and isopropanol and washed with 80% ethanol. Protein and RNA were removed using the QIAamp DNA stool Minikit (Qiagen) following the manufacturer's instructions. DNA samples were amplified across the V3-V4 region (Q5 polymerase; New England Biolabs) with these primers F: 5′-ACTCCTACGGGAGGCAGCAG-3′; R: 5′-GGACTACHVGGGTWTCTAAT-3′ and sequenced on an Illumina Miseq (2 × 300 bp). Data analysis was performed using QIIME 1.9.1 (28) using default parameters as described previously (29). Briefly, demultiplexed paired end data were quality filtered and paired using the Fastq-join algorithm with no errors allowed. Operational taxonomic units (OTUs) were picked using 97% similarity with UCLUST, and taxonomy was assigned with Greengenes database. The resulting OTU table was filtered by removing OTUs with <0.01% sequences and those relating to Cyanobacteria or Chloroplast. Further analysis was performed with R software (3.4.2). For statistical analysis, abundance data was transformed using the Hellinger method. Differences between treatment groups were determined by adonis (vegan 2.5-2) with 9999 permutations, alpha 0.05 and with the phyloseq package 1.25.2 (30) and Calypso 8.78 (31).

### Statistics

Mann–Whitney *U*-test was used for analysis of the differences between the mean of groups and Wilcoxon paired test for paired samples. For microbiota data, significant differences in the relative abundance of genus between treatment groups were determined by one-way ANOVA with *post-hoc* Tukey's test. Differences in overall microbial community between treatment groups were determined by adonis. *p* < 0.05 were considered statistically significant.

## Results

### Characterization of Food Grade TiO_2_ (E171)

We employed dynamic light scattering (DLS) to determine the hydrodynamic size of the E171 product used in this study. DLS revealed that the TiO_2_ nanoparticles dispersed in drinking water (5 mg/ml, pH 7.8) have an average hydrodynamic diameter of 367 nm, a polydispersity index of 0.258 and a zeta potential of −23.0 mV (±4.5 mV). We also employed nanoparticle tracking analysis (NTA) and scanning electron microscopy (SEM) to further investigate the size and shape of the TiO_2_ nanoparticles dispersed in drinking water. NTA (Supplementary Figure 1A) showed that the TiO_2_ nanoparticles are roughly spherical in shape and range in diameter from 28 to 1,158 nm. On a number basis, the particle size distribution has a mean of 202 nm and a mode of 138 nm and, on a weight basis, the particle size distribution has a mean of 363 nm and a mode of 428 nm. The average particle diameter determined by NTA on a weight basis (363 nm) is in good agreement with that determined by DLS (367 nm). SEM (Supplementary Figure 1B) confirmed that the TiO2 nanoparticles are roughly spherical in shape and revealed that they can be classified into essentially four groups (based on diameter)−300, 150–200, 100, and 30–50 nm—which is consistent with the particle size distribution (on a number basis) obtained by NTA. TiO2 was predominantly in anatase form as per manufacturer's description. This was verified using X-ray powder diffraction (data not shown).

### Impact of Oral Administration of Food Grade TiO_2_ on Gut Microbiota Composition

**Figure 1:**
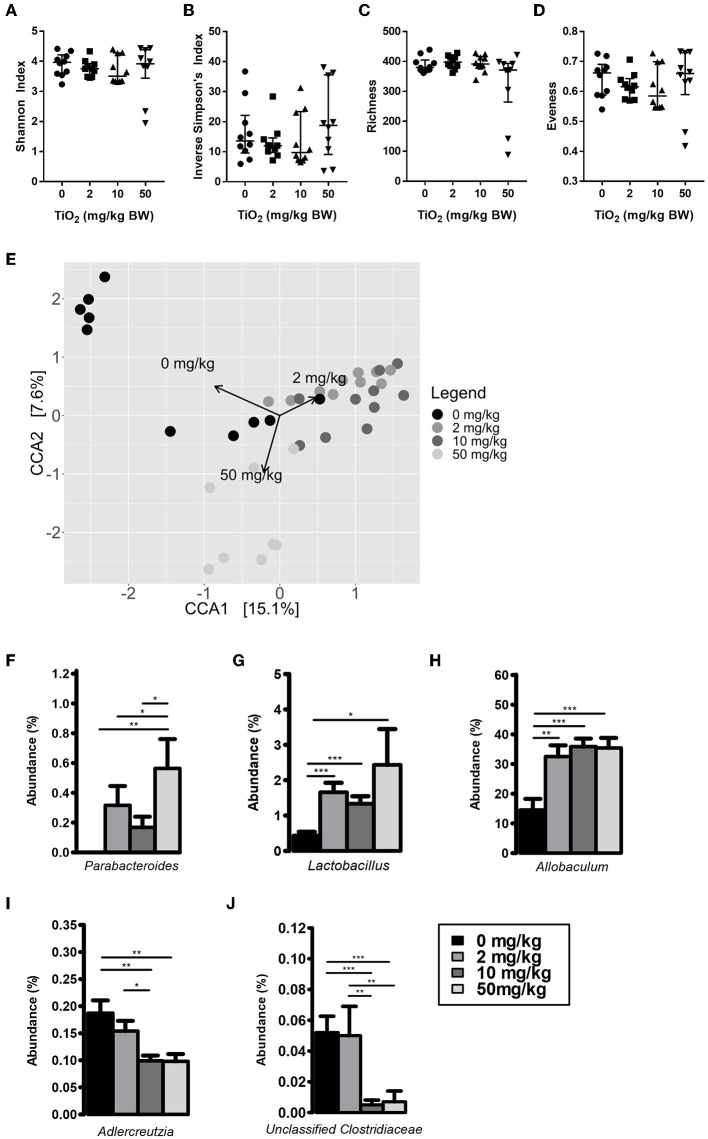
Impact of TiO_2_ on colonic microbiota composition. **(A–D)** Diversity of colonic microbiota composition of mice administered 0, 2, 10, or 50 mg TiO_2_/kg BW/day in drinking water was determined by **(A)** Shannon index, **(B)** Inverse Simpson's index, **(C)** richness, and **(D)** evenness (*n* = 10 mice per group from 2 cages of 5 mice). **(E)** Canonical correspondence analysis ordination of Bray-Curtis dissimilarity of colonic microbiota compositions of mice administered 0, 2, 10, or 50 mg TiO_2_/kg BW/day in drinking water. Ordination was constrained by dose of TiO_2_ and the arrows represent the doses of TiO_2_ driving the differences in microbiota composition observed. Composition differences between groups were significant as determined by adonis (*p* = 0.0012 for 0 vs. 2 mg TiO_2_/kg BW/day, *p* = 0.0006 for 0 vs. 10 mg TiO_2_/kg BW/day and *p* = 0.0105 for 0 vs. 50 mg TiO_2_/kg BW/day) (*n* = 10 mice per group from 2 cages of 5 mice). **(F**–**J)** Relative abundance of **(F)** *Parabacteroides* **(G)** *Lactobacillus*, **(H)** *Allobaculum*, **(I)** *Adlercreutzia*, and **(J)** Unclassified *Clostridiaceae* observed in colonic microbiota of mice administered 0, 2, 10, or 50 mg TiO_2_/kg BW/day in drinking water. *p < 0.05, **p < 0.01, ***p < 0.005 as determined by one-way ANOVA with *post-hoc* Tukey's test on Hellinger-transformed data (*n* = 10 mice per group from 2 cages of 5 mice).

We first determined whether exposure to TiO_2_ over a range of physiologically relevant doses impacted gut bacterial communities *in vivo*. To achieve this, mice were administered TiO_2_ via drinking water at doses of either 0, 2, 10, or 50 mg TiO_2_/kg BW/day for 3 weeks. Sequencing of the 16S rRNA gene from fecal samples revealed that TiO_2_ had limited effects on bacterial diversity as determined by Inverse Simpson and Shannon analyses (Figures 1A,B) nor bacterial richness (Figure 1C), evenness (Figure 1D) or Faith's diversity (Supplementary Figure 2A) at these doses. However, there was still a trend toward decrease in mice treated with physiological doses of TiO_2_ (2 and 10 mg TiO_2_/kg BW/day). On the other hand, both weighted (Supplementary Figure 2B) and unweighted UniFrac (Supplementary Figure 2C) principal coordinate analysis (PCoA) showed some clustering of bacterial composition in control vs. TiO_2_ treated mice. To test this further, we performed canonical correspondence analysis (CCA) constrained to the 4 distinct TiO_2_ concentrations used, which revealed significant clustering in bacterial composition driven by 2 mg TiO_2_/kg BW/day (*p* = 0.0011) and 50 mg TiO_2_/kg BW/day (*p* = 0.0123) TiO_2_ treatment (Figure 1E). We also performed CCA with TiO_2_ as a continuous variable, which reveals a dose dependent effect of TiO_2_ on microbiota composition (Supplementary Figure 2D). Treatment with TiO_2_ significantly affected gut microbiota composition independently of the cage effect (with overall treatment effect: *F*-value = 8.2407, *R*^2^ = 0.31644, Df = 3, *p* < 0.001 and impact of treatment corrected for the cage effect: *F*-value = 5.8511, *R*^2^ = 0.2996, Df = 3, *p* < 0.001 both by adonis). We then determined the impact of TiO_2_ at deeper levels and found significant changes at the genus level. *Parabacteroides* were significantly elevated in TiO_2_ treated mice, at a dose of 50 mg TiO_2_/kg BW/day (Figure 1F) while *Lactobacillus* and *Allobaculum* were significantly elevated at all doses tested (Figures 1G,H). On the other hand, *Adlercreutzia* (Figure 1I) and Unclassified *Clostridiaceae* (Figure 1J) were significantly decreased in the groups treated with TiO_2_ at the doses of 10 and 50 mg TiO_2_/kg BW/dayrelative to the untreated group. These results suggest that TiO_2_ had a minor impact on microbiota composition *in vivo*, while affecting few taxa at the genus level. The gut microbiota composition in the small intestine was also analyzed to determine whether TiO_2_ might have a greater effect here than in the colon. Bacterial diversity indices (Richness, evenness, Shannon, Inverse Simpson and Faith's diversity) were not significantly affected at doses of 10 and 50 mg TiO_2_/kg BW/day (Supplementary Figure 2E), although these trended toward decrease with increasing dose of TiO_2_. Unlike in the colon, TiO_2_ did not significantly alter the small intestine bacterial composition (*p* > 0.05 by adonis) and weighted and unweighted UniFrac PCoA analysis revealed no obvious clustering (Supplementary Figures 2F,G). Overall, TiO_2_ did not appear to dramatically impact on small intestinal microbiota composition. We also performed co-occurrence analysis by examining microbial interactions from mice treated with either 0, 2, 10, or 50 mg TiO_2_/kg BW/day. We found that certain genera are consistently associated with each other regardless of TiO_2_ treatment (*Ruminococcus, Desulfovibrio*, and *Oscillospira* are positively connected). Increasing TiO_2_ intake, especially at the dose of 10 and 50 mg/kg BW/day resulted in more significant connections within the network, as well as increased number of genera with significant contributions. For example, while Akkermansia was not significantly involved in the microbial network of mice administered 0, 2, or 10 mg TiO_2_/kg BW/day, it is involved at a dose of 50 mg/kg involving numerous co-exclusion relationships. These co-occurrence graphs are presented in Supplementary Figures 2H–K. These results were verified using the deblur pipeline (32) which resolves amplicon sequences much more accurately (Supplementary Figures 3A–F).

### Food Grade TiO_2_ Modulates Commensal Bacterial Activity

**Figure 2:**
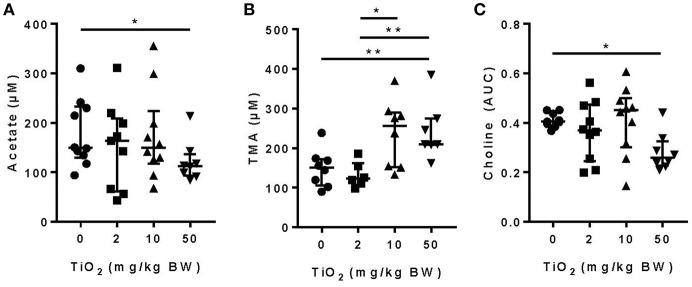
Impact of TiO_2_ treatment on gut bacterial metabolites. **(A,B)** Concentrations of **(A)** the SCFA acetate and **(B)** TMA were determined NMR on the serum of mice administered 0, 2, 10, or 50 mg TiO_2_/kg BW/day in drinking water. Data is represented as median ± interquartile range (IQR). *p < 0.05 as determined by Mann–Whitney *U*-test (*n* = 10 mice per group). **(C)** Concentration of choline was determined by liquid-chromatography mass spectrometry in plasma of fasted mice treated with 0, 2, 10, or 50 mg TiO_2_/kg BW/day in drinking water. Concentration is represented as area under curve (AUC). Data are represented as median ± IQR. *p < 0.05 as determined by Mann–Whitney *U*-test (*n* = 10 mice per group). **p < 0.01.

We and others have shown that gut bacterial metabolites such as SCFAs can have a dramatic impact on host immune function and disease development (1–5, 33, 34). Mice treated with 50 mg TiO_2_/kg BW/day had a significant decrease in the SCFA, acetate, in the plasma, suggesting a possible impact of TiO_2_ on host-bacterial interaction (Figure 2A). Such effects on bacterial metabolites were not limited to SCFAs as TMA, a bacterial product associated with development of atherosclerosis (35), was increased at doses of 10 and 50 mg TiO_2_/kg BW/day (Figure 2B). TMA is a product of conversion of choline, which was also found to be decreased at 50 mg TiO_2_/kg BW/day (Figure 2C), suggesting that increased TMA was not due to a change in the substrate availability but potentially changes in bacterial activity.

### Food Grade TiO_2_ Promotes the Cluster of Commensal Bacteria and Biofilm Formation

**Figure 3:**
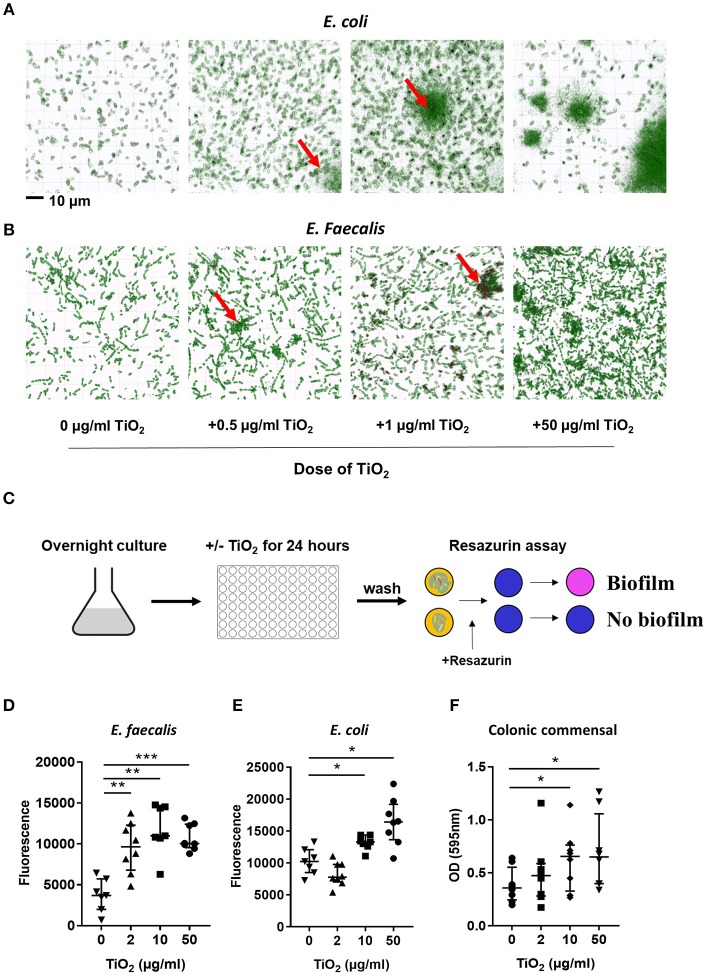
TiO_2_ triggers biofilm formation by commensal bacteria. **(A,B)** The clustering effect of TiO_2_ on **(A)** *E. coli* and **(B)** *E. faecalis in vitro* was visualized by Nanolive imaging in the presence of 0, 0.5, 1, or 50 μg/ml TiO_2_ after 24 h incubation. False-coloring was applied to images based on refractive index, where black represents the refractive index of TiO_2_ and green represents bacteria. **(C)** Schematic representation of biofilm formation assay and resazurin viability assay to assess biofilm formation *in vitro*. **(D,E)** The ability of *E. faecalis* and *E. coli* to form biofilm *in vitro* in the presence of 0, 2, 10, or 50 μg/ml TiO_2_ in culture (24 and 72 h, respectively) was assessed by the resazurin viability assay (*n* = 8 replicates). **(F)** Colonic bacteria were isolated and biofilm formation assessed in the presence of 0, 2, 10, or 50 μg/ml TiO_2_ in culture after 5 days (*n* = 6 mice per group). Data are represented as median ± IQR. *p < 0.05, **p < 0.01; ***p < 0.001 as determined by Wilcoxon paired test compared to non-treated group.

Bacteria also communicate with the host via direct interactions. Studies have shown that attachment of biofilm on the colonic epithelium was correlated with colorectal cancer, a disease in which TiO_2_ has aggravating effects (36). To explore the possibility that TiO_2_ might promote biofilm formation, we incubated two types of commensal bacteria, *E. coli* and *E. faecalis*, in the presence of TiO_2_. Nanolive imaging revealed the clustering effect of TiO_2_ on both *E. coli* (Figure 3A) and *E. faecalis* (Figure 3B) *in vitro* in a dose dependent manner. To determine whether the cluster of bacteria was due to biofilm formation, we performed *in vitro* culture of either *E. faecalis or E. coli* in the presence of 2, 10, or 50 μg/ml of TiO_2_ for 24 or 72 h, respectively. Using the resazurin viability assay (Figure 3C), we found that TiO_2_ treatment significantly increased biofilm formation in both subsets of bacteria (Figures 3D,E) but not in *Staphylococcus epidermidis*, a strain known for its inability to form biofilm (Supplementary Figure 4). We confirmed by confocal microscopy that TiO_2_ treatment increased biofilm formation in both *E. coli* and *E. faecalis* (Supplementary Figure 5). To determine whether such effects were applicable to bacteria in the complex environment of the gut microbiota, we incubated commensal bacteria derived from mouse colons anaerobically for 5 days with doses of 2, 10, and 50 μg/ml of TiO_2_. Both the doses of 10 and 50 μg/ml TiO_2_ significantly promoted biofilm formation by commensal bacteria (Figure 3F). These data highlight that TiO_2_ can affect the spatial organization of the gut microbiota and thus its potential interaction with the host.

### TiO_2_ Affects Colonic Epithelial Function

**Figure 4:**
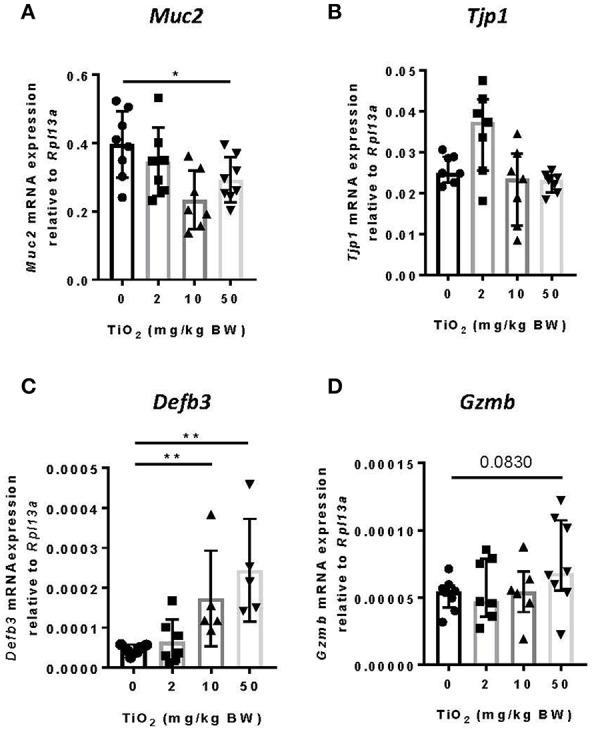
TiO_2_ impairs colonic epithelial function. **(A–D)** The impact of TiO_2_ on colonic epithelial function was determined by comparison of gene expression of key markers **(A)** *Muc2*, **(B)** *Tjp1*, **(C)** *Defb3*, and **(D)** *Gzmb in* colonic tissue of mice administered 0, 2, 10, or 50 mg TiO_2_/kg BW/day in drinking water (*n* = 5–8 mice per group). Data are represented as median ± IQR. *p < 0.05, **p < 0.01 as determined by Mann–Whitney *U*-test.

While the impact of biofilm formation on the host is unclear, impaired mucus production has been correlated with the presence of bacterial biofilms (11). To determine whether TiO_2_ might impact the mucus layer, we examined colonic *Muc2* gene expression in the colon. We found that both 10 and 50 mg TiO_2_/kg BW/day decreased *Muc2* expression, suggesting a detrimental impact of TiO_2_ on the mucus layer (Figure 4A). While biofilm formation has been reported in colitis and colorectal cancer (36), these diseases have also been linked to increased gut permeability (37). To test whether TiO_2_ affects gut permeability, we studied the expression of *Tjp1* (encoding for zonula occludens 1), which was unchanged by TiO_2_ treatment (Figure 4B), suggesting no impact of TiO_2_ on gut permeability. The other major mechanism of bacterial exclusion is through the release of antimicrobial peptides. Beta defensin is expressed predominantly in the colon and we found that *Defb3* (encoding for beta-defensin-3) was elevated by treatment at doses of both 10 and 50 mg TiO_2_/kg BW/day (Figure 4C). Expressions of other antimicrobial peptides such as granzyme B (Figure 4D), cathelin-related antimicrobial peptide (CRAMP), regenerating islet-derived protein 3 gamma (REG3 gamma) and p-lysozyme (PLYz) (Supplementary Figure 6) were unchanged. Therefore, TiO_2_ treatment impairs the expression of key colonic epithelial factors involved in gut homeostasis.

### TiO_2_ Contributes to Increased Colonic Macrophages and Associated Cytokines

**Figure 5:**
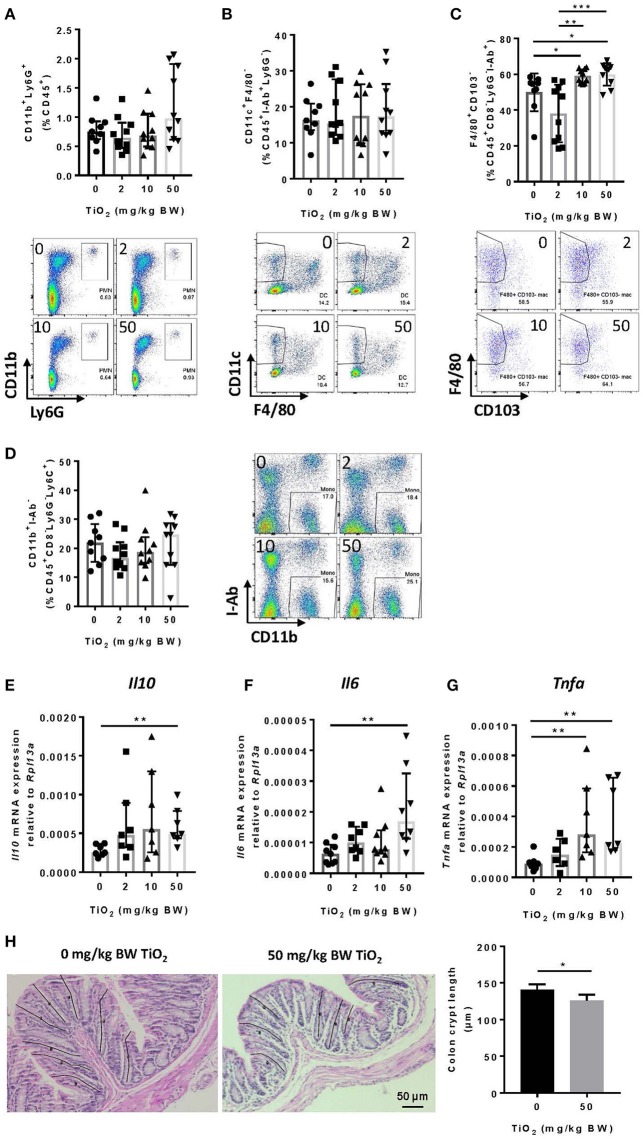
TiO_2_ promotes colonic inflammation. **(A–D)** Proportion of the colonic immune subsets **(A)** neutrophils **(B)** dendritic cells, **(C)** macrophages, and **(D)** monocytes in mice administered 0, 2, 10, or 50 mg TiO_2_/kg BW/day in drinking water, were determined by flow cytometric analysis (*n* = 8–10 mice per group). **(E–G)** Expression of genes encoding for **(E)** IL-10, **(F)** IL-6, and **(G)** TNF-α was determined by qPCR from colonic tissue of mice treated with 0, 2, 10, or 50 mg TiO_2_/kg BW/day in drinking water (*n* = 10 mice per group). **(H)** (Left—images) H&E stained colonic tissue section was evaluated for crypt length changes in 0 vs. 50 mg TiO_2_/kg BW/day. Representative histology images are shown for each group, black lines represent representative crypt length measurements (*n* = 5 mice per group). (Right—Graph) Quantification of colonic crypt length in untreated mice vs. mice treated with 50 mg TiO_2_/kg BW/day. Data are represented as median ± IQR. *p < 0.05, **p < 0.01 as determined by Mann–Whitney *U*-test. ***p < 0.001.

Decreased *Muc2* has been correlated with inflammation and MUC2 deficiency leads to spontaneous colitis (38). To test whether TiO_2_ might affect innate immune cells in the colon, we studied myeloid immune cell populations by flow cytometry. While neutrophils (CD45^+^Ly6g^+^CD11b^+^) (Figure 5A) and dendritic cells (CD45^+^I-ab^+^Ly6g^−^F4/80^−^CD11c^+^) were unchanged (Figure 5B), macrophages (CD45^+^F4/80^+^CD8^−^Ly6g^−^I-ab^+^CD11b^+^ CD103^−^) were significantly increased by TiO_2_ at 10 and 50 mg TiO_2_/kg BW/day (Figure 5C). This change was not due to an increased recruitment of total monocytes (CD45^+^CD8^−^Ly6G^−^Ly6C^+^CD11b^+^I-ab^−^) (Figure 5D), suggesting a potential *in situ* proliferation of macrophages (gating strategies shown in Supplementary Figure 7). Colonic macrophages are a major source of IL-6, TNF-alpha and IL-10, cytokines, which were also upregulated in the colon of TiO_2_ treated mice (Figures 5E–G). We also observed a significant reduction in colonic crypt length by histological analysis of mice treated with 50 mg TiO_2_/kg BW/day (Figure 5H) while colon length was unchanged (data not shown). Thus, TiO_2_ treatment triggers changes in the colonic myeloid compartment as well as structural changes in the colon.

### TiO_2_ Promotes Increased CD8^+^ T Cell Infiltration in the Colon and Increased Inflammatory Cytokines

**Figure 6:**
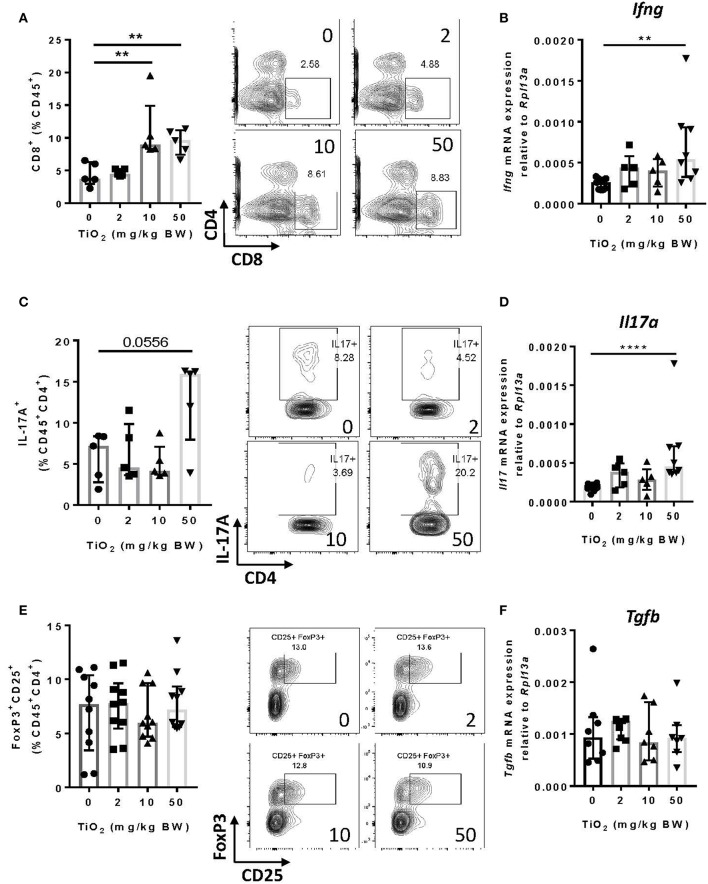
TiO_2_ treatment results in adaptive immune cell infiltration into the colon. **(A)** Proportion of colonic CD8^+^ T cells was quantified by flow cytometric analysis on mice treated with 0, 2, 10, or 50 mg TiO_2_/kg BW/day in drinking water (left); representative gating is shown (right) (*n* = 5 mice per group). **(B)** Expression of the gene encoding for IFN-γ was determined by qPCR on colon tissue of mice treated with 0, 2, 10, or 50 mg TiO_2_/kg BW/day in drinking water (*n* = 6–8 mice per group). **(C)** Proportion of IL-17A producing CD4 T cells from the colon was determined by flow cytometric analysis on mice treated with 0, 2, 10, or 50 mg TiO_2_/kg BW/day in drinking water (left); representative gating is shown (right) (*n* = 5 mice per group). **(D)** Expression of the gene encoding for IL-17A was determined by qPCR on colon tissue of mice treated with 0, 2, 10, or 50 mg TiO_2_/kg BW/day in drinking water (*n* = 6–8 mice per group). **(E)** Proportion of colonic regulatory T cells was determined by flow cytometric analysis on mice treated with 0, 2, 10, or 50 mg TiO_2_/kg BW/day in drinking water (left); representative gating is shown (right) (*n* = 8 mice per group). **(F)** Expression of the gene encoding for TGF-beta was determined by qPCR on colon tissue of mice treated with 0, 2, 10 or 50 mg TiO_2_/kg BW/day in drinking water (*n* = 6–8 mice per group). Data are represented as median ± IQR. **p < 0.01, ****p < 0.001 as determined by Mann–Whitney *U*-test.

Other cell subsets can produce TNF-alpha, particularly CD8^+^ T cells (39). By flow cytometry analysis, we found that CD8^+^ T cells were significantly increased from 10 mg TiO_2_/kg BW/day treatment (Figure 6A), as was expression of interferon-gamma in this cell subset (Figure 6B). Increased proportions of both macrophages and CD8^+^ T cells suggest a state of colonic inflammation in TiO_2_ treated mice which is consistent with the increased proportion of colonic Th17 cells (*p* = 0.0556) (Figure 6C) as well as significantly increased expression of IL-17A (Figure 6D). On the other hand, neither regulatory T cells (Figure 6E) nor TGF-beta (Figure 6F) were affected by TiO_2_ treatment. Gating strategies for flow cytometry analysis are shown in Supplementary Figure 8. These findings show that TiO_2_ treatment impairs immune homeostasis in the colon and promotes an inflammatory environment.

## Discussion

The ubiquitous use and daily consumption of TiO_2_ by the general population warrants investigation into its potential impact on health. After only a few weeks of daily TiO_2_ consumption, we observed that colonic homeostasis was significantly impaired in mice. While TiO_2_ impacted bacterial function by causing changes in bacterial metabolites (acetate and TMA) and by promoting biofilm formation by commensal bacteria, TiO_2_ had minimal impact on gut microbiota composition. One of the major mechanisms of physical separation between host and gut bacteria was impaired by TiO_2_, as shown by decreased *Muc2* expression and increased *Defb3* expression in colonic epithelial cells. We also observed increased macrophages, CD8^+^ T cells and Th17 T cells as well as increased inflammatory cytokines in the colon. This increased inflammation was associated with decreased colonic crypt length, as reported in inflammatory bowel diseases (40). Disruption of gut homeostasis due to chronic exposure to TiO_2_ may thus prime the host for conditions such as inflammatory bowel diseases or colorectal cancer.

Consumption of TiO_2_ had no impact on microbiota diversity in either the small intestine or colon. Using a constrained analysis, we found that microbiota composition in the small intestine was unchanged while some colonic microbiota changes were driven by 2 and 50 mg TiO_2_/kg BW/day. However, only a few taxa at the genus level were significantly altered in the colon, suggesting that TiO_2_ consumption is associated with minor changes in bacterial communities. Similarly, TiO_2_ might not dramatically reshape the human microbiota *in vivo* which would confirm previous *in vitro* findings in a model of simplified human microbiota (41, 42). However, treatment with TiO_2_ over a longer period of time, as previously done by treating mice for 12 weeks with emulsifiers polysorbate-80 (P80) and carboxylmethyl cellulose (CMC), might have a more dramatic impact (43).

The impact of TiO_2_ on gut microbiota at the genus level shared some similarities with this study on emulsifiers in which mice treated with CMC had a significant increase in *Lactobacillus* and *Allobacullum* (43). The increase in *Lactobacillus* is particularly interesting as these bacteria are a major biofilm producer, suggesting that TiO_2_ might favor the growth of biofilm producing bacteria. Another study suggests that TiO_2_ may enhance the growth of *Lactobacillus* (44). Previous studies have shown that TiO_2_ could either bind onto the surface of bacteria or bacteria could uptake TiO_2_ (45), which might trigger a defense mechanism contributing to biofilm formation as we obser*ved in vitro*. We also found that TiO_2_ mediated changes in the gut environment, such as decreased *Muc2* expression, which have been shown to favor biofilm formation. Since bacterially derived SCFAs have been shown to promote mucus layer thickness, decreased acetate at the dose of TiO_2_ of 50 mg TiO_2_/kg BW/day could partially explained changes in mucus gene expression in mice treated at this dose of TiO_2_. TiO_2_ might also directly affect the function of mucus-producing goblet cells, as a previous report suggests an efficient uptake of TiO_2_ by goblet cells *in vitro* (46). The mucus layer is an efficient physical barrier preventing bacterial attachment to the epithelium and so its impairment by TiO_2_ might thus favor bacterial attachment and biofilm formation in the gut. Similarly, emulsifiers have been shown to decrease the mucus layer leading to closer contact between commensal bacteria and the epithelium (43). However, whether emulsifiers might favor biofilm formation is unknown. While we did not observe any impact of TiO_2_ on gut permeability related genes, *Defb3* was upregulated which might be a compensatory mechanism to control the interaction with the commensal bacteria.

In the colonic lamina propria, we observed a significant impact of TiO_2_ on both innate and adaptive immune cells with increased macrophages, Th17 and CD8^+^ T cells. This proinflammatory effect of TiO_2_ is confirmed by changes in the cytokine environment with increased IL-6, IL-17, and TNF-alpha gene expression as well as decreased colonic crypt length. The later has also been reported in rats treated for 100 days with 10 mg TiO_2_/kg BW/day (19). Our findings suggest that some of the changes induced by TiO_2_ occur after as little as 30 days of daily TiO_2_ treatment.

In summary, our findings demonstrate that TiO_2_ profoundly affects gut homeostasis in mice and that such changes can occur over a period of time significantly shorter than the exposure typical for the human population. These changes were most significant at the highest dose of 50 mg TiO_2_/kg BW/day, but still significant at the physiological doses of 2 and 10 mg TiO_2_/kg BW/day. The pro-inflammatory environment and biofilm formation induced by TiO_2_ predispose the host to conditions such as inflammatory bowel diseases and colorectal cancer, both of which have been shown to be aggravated by TiO_2_ (19, 20). The reduced SCFA production at the highest dose of TiO_2_ has profound health implications as acetate has been shown to provide protection from colitis, colorectal cancer, food allergy, asthma and type 1 diabetes (2–5).

Finally, this work highlights the need for further research into how TiO_2_, on its own and in combination with other food additives, affects human health. Such research would better inform the regulation of food additives such TiO_2_ and thus potentially reduce the incidence of non-communicable diseases associated with the western lifestyle.

## Ethics Statement

All experimental procedures involving animals were approved by the University of Sydney Animal Ethics Committee under protocol number 2014/696.

## Author Contributions

GP and JT performed most of the experiments and analysis and wrote the manuscript. BJ and NK did the experiments related to biofilm and contributed to the manuscript writing. AA did the NMR and associated analysis and did the NTA for the TiO_2_ characterization. JO and YK did the mass spectrometry and associated analysis. FS contributed to the animal work, immune analysis, and TiO_2_ characterization and provided input throughout the project. JD did the SEM for the TiO_2_ characterization. SD did the CLSM. DK helped with the nanolive imaging. RM did the microbiota sequencing. DS did the microbiota sequencing and bioinformatic analysis. WC conceived the idea to study the impact of TiO_2_ on microbiota composition, and assisted with the TiO_2_ characterization. LM is the chief investigator in all the funding that supported this project, conceived the idea to study the impact of TiO_2_ on gut homeostasis and biofilm formation, designed and supervised the project, participated in the experiments and wrote the manuscript. All authors reviewed the manuscript.

### Conflict of Interest Statement

The authors declare that the research was conducted in the absence of any commercial or financial relationships that could be construed as a potential conflict of interest.
