# Corrigendum: Impact of the Food Additive Titanium Dioxide (E171) on Gut Microbiota-Host Interaction

**DOI:** 10.3389/fnut.2019.00100

**Published:** 2019-07-02

**Authors:** Gabriela Pinget, Jian Tan, Bartlomiej Janac, Nadeem O. Kaakoush, Alexandra Sophie Angelatos, John O'Sullivan, Yen Chin Koay, Frederic Sierro, Joel Davis, Shiva Kamini Divakarla, Dipesh Khanal, Robert J. Moore, Dragana Stanley, Wojciech Chrzanowski, Laurence Macia

**Affiliations:** ^1^The Charles Perkins Centre, The University of Sydney, Sydney, NSW, Australia; ^2^Faculty of Medicine and Health, School of Medical Sciences, The University of Sydney, Sydney, NSW, Australia; ^3^Sydney Nano Institute, The University of Sydney, Sydney, NSW, Australia; ^4^Human Health, Nuclear Science & Technology and Landmark Infrastructure (NSTLI), Australian Nuclear Science and Technology Organisation, Sydney, NSW, Australia; ^5^School of Medical Sciences, University of New South Wales, Sydney, NSW, Australia; ^6^Department of Cardiology, Charles Perkins Centre, Royal Prince Alfred Hospital, Heart Research Institute, University of Sydney, Sydney, NSW, Australia; ^7^Sydney Pharmacy School, The University of Sydney, Sydney, NSW, Australia; ^8^School of Science, RMIT University, Bundoora, VIC, Australia; ^9^School of Health, Medical and Applied Sciences, Central Queensland University, Rockhampton, QLD, Australia

**Keywords:** biofilm, gut microbiota, immune cells, inflammation, titanium dioxide

In the original article, there was an error. In the Materials and Methods section, there was an error concerning the supplier of E171.

A correction has been made to the **Materials and Methods** section, subsection **E171 Characterization, Size and Morphology**:

“Food grade TiO_2_ was purchased from All Color Supplies PTY. Average hydrodynamic diameter, polydispersity index and zeta potential of the TiO_2_ nanoparticles dispersed in drinking water were determined with a Malvern Zetasizer Nano ZS at 25°C. The dispersion was measured 3 times for both size and zeta potential. The size distribution and shape of the TiO_2_ nanoparticles dispersed in mice drinking water were determined using a NanoSight NS300 (equipped with a sCMOS camera) at 25°C. The dispersion was measured 5 times (1 min per measurement). The size distribution and shape of the TiO_2_ nanoparticles dispersed in drinking water were further investigated using a Zeiss Ultra Plus scanning electron microscope operated at an accelerating voltage of 10 kV. A drop of the nanoparticle dispersion was allowed to dry on a stub, after which ~20 Å of platinum metal was sputter coated onto the stub under vacuum to prevent charging.”

The authors apologize for this error and state that this does not change the scientific conclusions of the article in any way. The original article has been updated.

