# Supplementary material for: Impact of the Food Additive Titanium Dioxide (E171) on Gut Microbiota-Host Interaction
Source: Front Nutr. 2019 May 14;6:57. doi: 10.3389/fnut.2019.00057 (PMC6534185; doi:10.3389/fnut.2019.00057)
Supplement: Supplementary file 1 [file Data_Sheet_1.pdf]

Supplementary Information

Impact of the food additive titanium dioxide (E171) on gut microbiota-host interaction

Gabriela Pinget, Jian Tan, Bartlomiej Janac, Nadeem O. Kaakoush, Alexandra Sophie Angelatos, John O'Sullivan, Yen Chin Koay, Frederic Sierro, Joel Davis, Shiva Kamini Divakarla, Dipesh Khanal, Robert Moore, Dragana Stanley, Wojciech Chrzanowski, Laurence Macia

SI Materials and Methods

Antibodies used for flow cytometry analysis

Cell suspensions were stained for FACS analysis using the following anti-mouse conjugate antibodies: IL-17A PE (TC11-18H10.1), CD3e AF488 (17A2), CD4 PerCP/Cy5.5, (GK1.5), CD45 BV785 (30-F11), CD25 BV605 (PC61), CD11c FITC (N418), CD11b APC/Cy7 (M1/70), Ly6G AF647 (1A8), Ly6C BV605 (HK1.4), I-Ab Pacific Blue (AF6-120.1), F4/80 PE/Cy7 (BM8) and CD8a BV711 (536.7) purchased from Biolegend and FoxP3 APC (REA788) purchased from Miltenyi Biotec.

Primer sequences

Table 1. Primers used in qPCR assays

Gene	NCBI ref sequence	Sequence
Camp	NM_009921.2	F’ CTTCAACCAGCAGTCCCTAGACA<br>R’ TCCAGGTCCAGGAGACGGTA
Cyp1a1	NM_001136059.2	F’ CCCACAGCACCACAAGAGAT<br>R’ ACCTGCCACTGGTTCACAAA
Defb3	NM_013756.2	F’ GCATTGGCAACACTCGTCAGA<br>R′ CGGGATCTTGGTCTTCTCTA
Gzmb	NM_013542.2	F’ GCTGCTCACTGTGAAGGAAGTAT<br>R’ GGGATGACTTGCTGGGTCTT
Ifng	NM_008337.4	F’ GCAAAAGGATGGTGACATGA<br>R’ TTCGCCTTGCTGTTGCTGA
Il10	NM_010548.2	F’ AAGGGTTACTTGGGTTGCCA<br>R’ AAATCGATGACAGCGCCTCAG
Il17a	NM_010552	F’ ACGTTTCTCAGCAAACTTAC<br>R’CCCTTTACACCTTCTTTTCA
Il6	NM_031168.2	F’ CCTCTCTGCAAGAGACTTCCAT<br>R’ AGTCTCCTCTCCGGACTTGT
Lyz1	NM_013590.4	F’ CTGTGGGATCAATTGCAGTG<br>R’ TGGGACAGATCTCGGTTTTGAC
Muc2	NM_023566.3	F’ ATTCTGAAGCCTGGGGAGAT<br>R’GAAGTCGGGACAGGTGATGT
Reg3g	NM_011260	F’ AACAGAGGTGGATGGGAGTG<br>R’ GTGATTGCCTGAGGAAGAGG
Rpl13a	NM_009438	F‘ ATCCCTCCACCCTATGACAA<br>R’ GCCCCAGGTAAGCAAACTT
Tgfb1	NM_011577.1	F’ GGATACCAACTATTGCTTCAG<br>R’ TGTCCAGGCTCCAAATATAG
Tjp1	NM_001163574.1	F’ TCTGAGGGGAAGGCGGATGGTGCT<br>R’TTGTGGCTGCGCTTGTGGTGAGTAA
Tnfa	NM_013693.3	F’ ATGGCCTCCCTCTCATCAGT<br>R’ GTTTGCTACGACGTGGGCTA

Confocal microscopy and analysis of biofilm formation

E. coli or *E. faecalis* was grown in Trypticase Soy Broth (TSB) for a period of 16 hours at 37°C and 150 rpm. A 1:10 dilution of the resulting bacterial suspension was made in TSB for inoculation of the surfaces. Briefly, TiO_2 treated samples were made to concentration in 1 mL of the diluted bacterial suspension and biofilm was grown on microscope slides (placed in petri dishes containing water droplets to prevent moisture loss) over a period of seven days, with 0.1X TSB media (diluted in Milli-Q water) being replaced on alternate days to promote biofilm growth (as biofilm grows in response to environmental stresses) and to ensure sufficient nutrient availability for bacterial growth. On Day 10, media was removed and the samples were rinsed in 1X PBS in triplicate to remove any loosely attached bacteria from the surface.

Biofilms were stained using the fluorescent nucleic acid stain SYTO-9 for detection of live cells and the cell-impairment nucleic acid stain Propidium iodide (PI) for detection of the dead cells (i.e., cells with compromised membranes) (LIVE/DEAD Bac-Light Bacterial Viability Kit, Molecular Probes). The stain mixtures were prepared as per manufacturer instructions and added directly to the surfaces, and then allowed to incubate in the dark at room temperature for a period of 30 minutes prior to imaging using Confocal Laser Scanning Microscopy (CLSM). Live/dead stained biofilm samples were imaged using the Nikon C2 Confocal microscope with laser settings of 473 nm and 559 nm for green (live) and red (dead) staining, respectively. Fiji ImageJ software was employed to generate images (using the Volume viewer function).

16S rRNA Gene Amplicon Sequencing Bioinformatics

To validate microbiome analysis, raw sequencing data was denoised using the deblur algorithm to allow sequences at be accurately resolved at a single-nucleotide level. Briefly, 16S rRNA gene sequencing data were cleaned of barcodes and primers and demultiplexed using tools available from the QIIME 1.9.1 package. Sequences were then trimmed to 400 nucleotide length (sequences shorter than 400 bp were discarded) and inputted into the deblur pipeline (v1.1.0-0) where sequences are dereplicated, singleton removed, sequence filtered and de novo chimeras removed. Taxonomy were assigned using the RDP training set (version 16) and a biom table generated. Exploration of data was performed using the R phyloseq package and Calypso as described in the main text.

Supplementary Figure 1

A

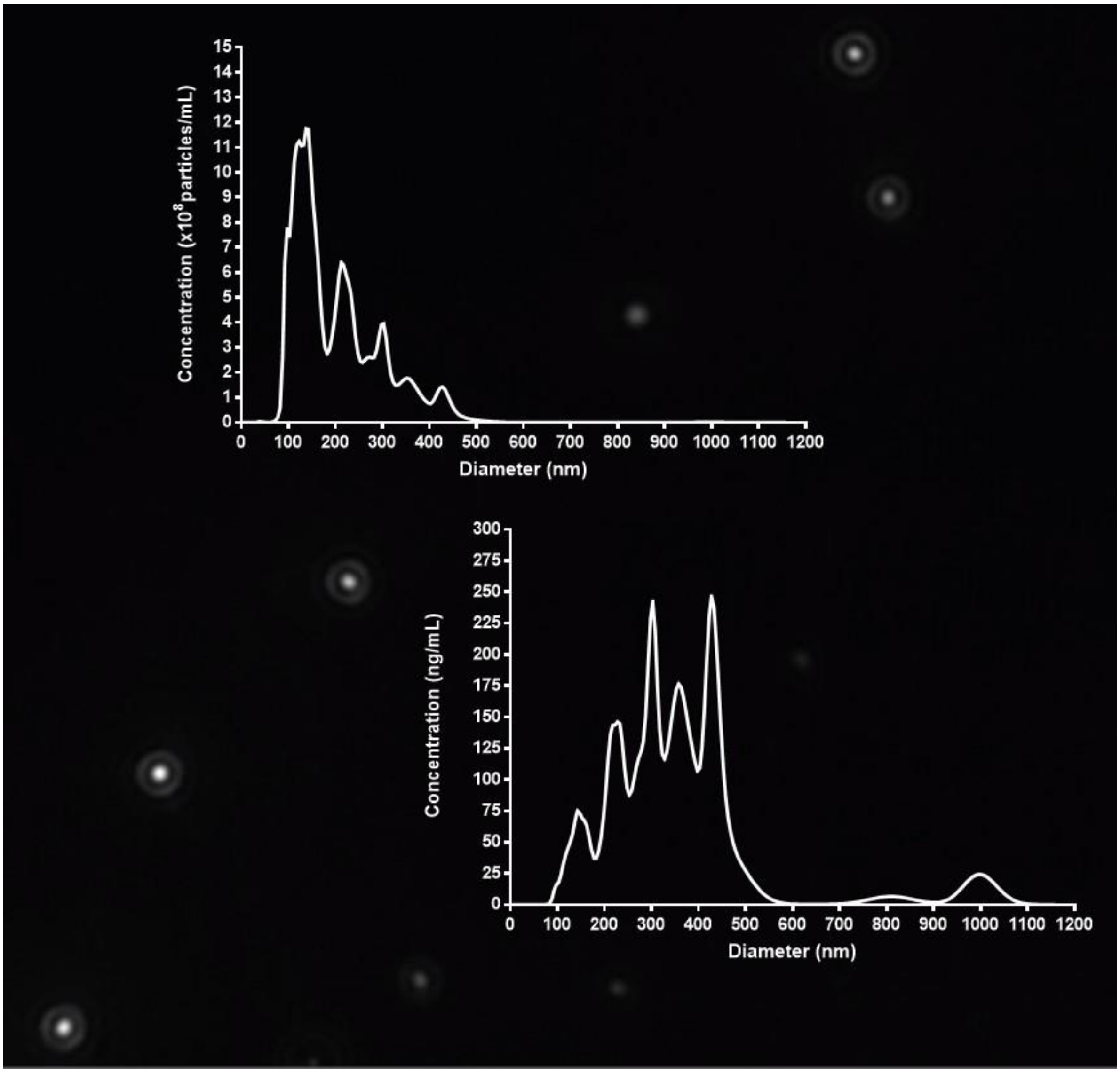

B

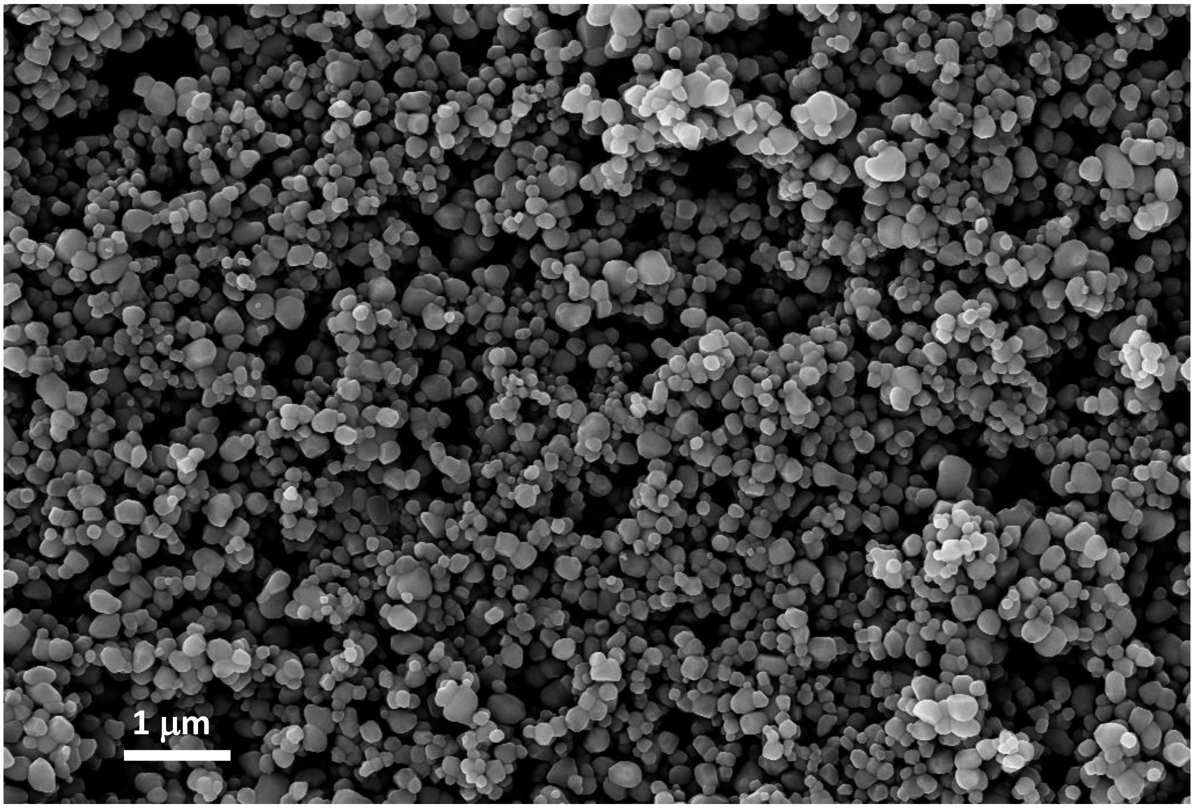

Supplementary Figure 1: Characterisation of the TiO_2 nanoparticles

(A) NTA revealed that the TiO_2 nanoparticles range in diameter from 28 nm to 1158 nm. The size distribution on a number basis (upper graph) has a mean of 202 nm and a mode of 138 nm. The size distribution on a weight basis (lower graph) has a mean of 363 nm and a mode of 428 nm. The average standard error associated with each diameter value is $\pm$10 nm. NTA further revealed that the nanoparticles are roughly spherical in shape, as shown in the image upon which the graphs are superimposed (which was obtained from a video recorded during the NTA). **(B)** An SEM image of the TiO_2 nanoparticles (10,000x magnification). SEM confirmed that the TiO2 nanoparticles are roughly spherical in shape and revealed that they can be classified into essentially four groups (based on diameter) – 300 nm, 150-200 nm, 100 nm and 30-50 nm.

Supplementary Figure 2 related to figure 1

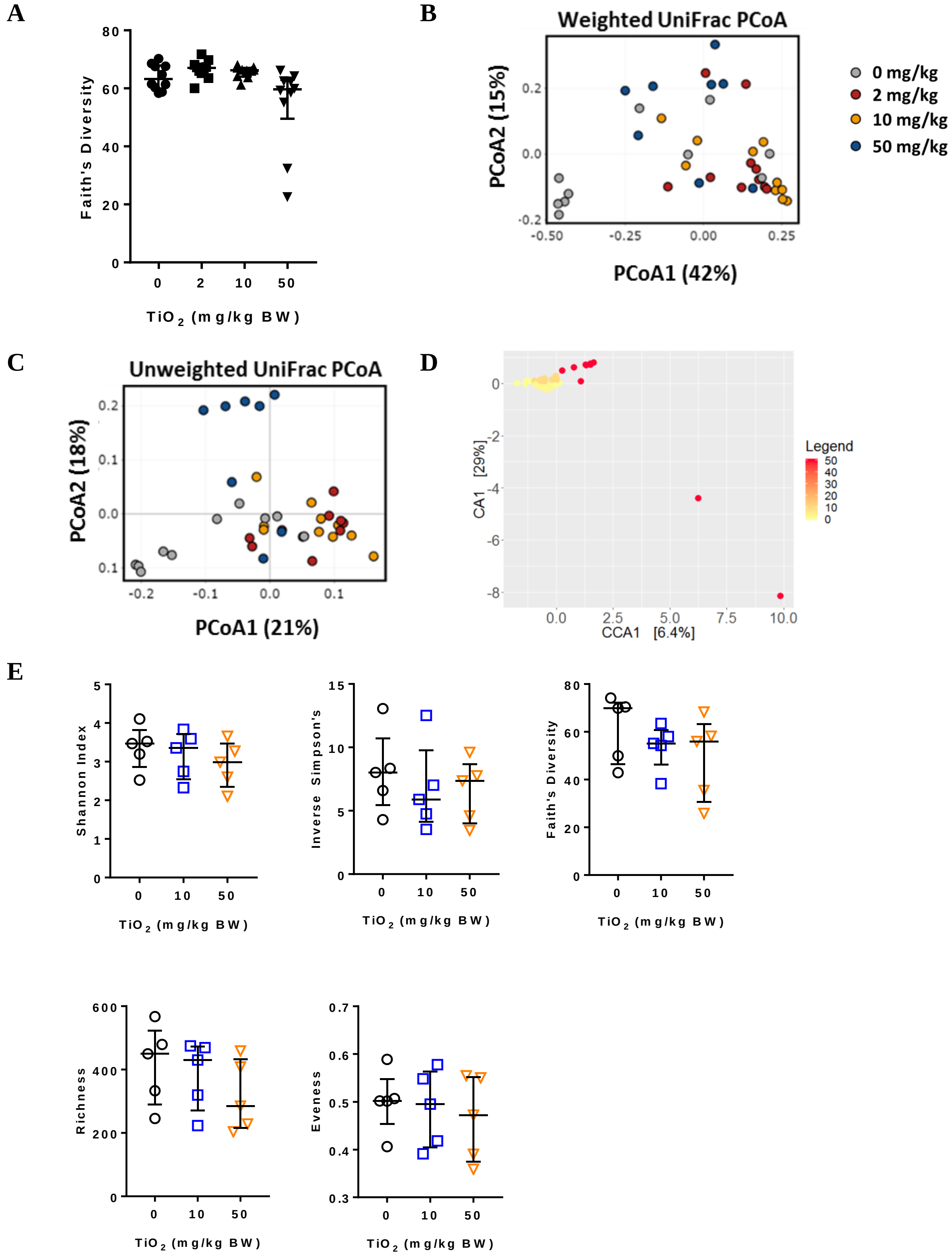

F

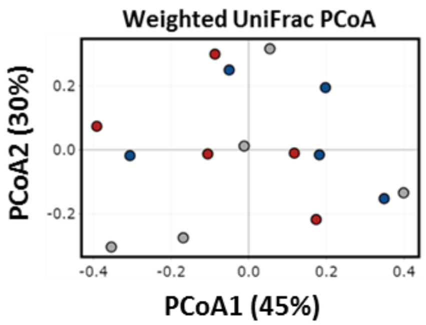
Weighted UniFrac PCoA
PCoA2 (30%)
PCoA1 (45%)
0.2
0.0
-0.2
-0.4
-0.2
0.0
0.2
0.4

G

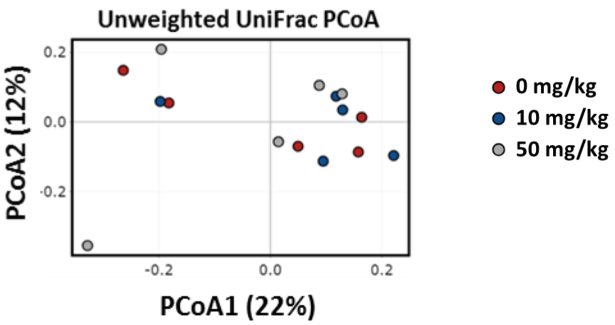
Unweighted UniFrac PCoA
PCoA2 (12%)
PCoA1 (22%)
0.2
0.0
-0.2
-0.2
0.0
0.2
0 mg/kg
10 mg/kg
50 mg/kg

H

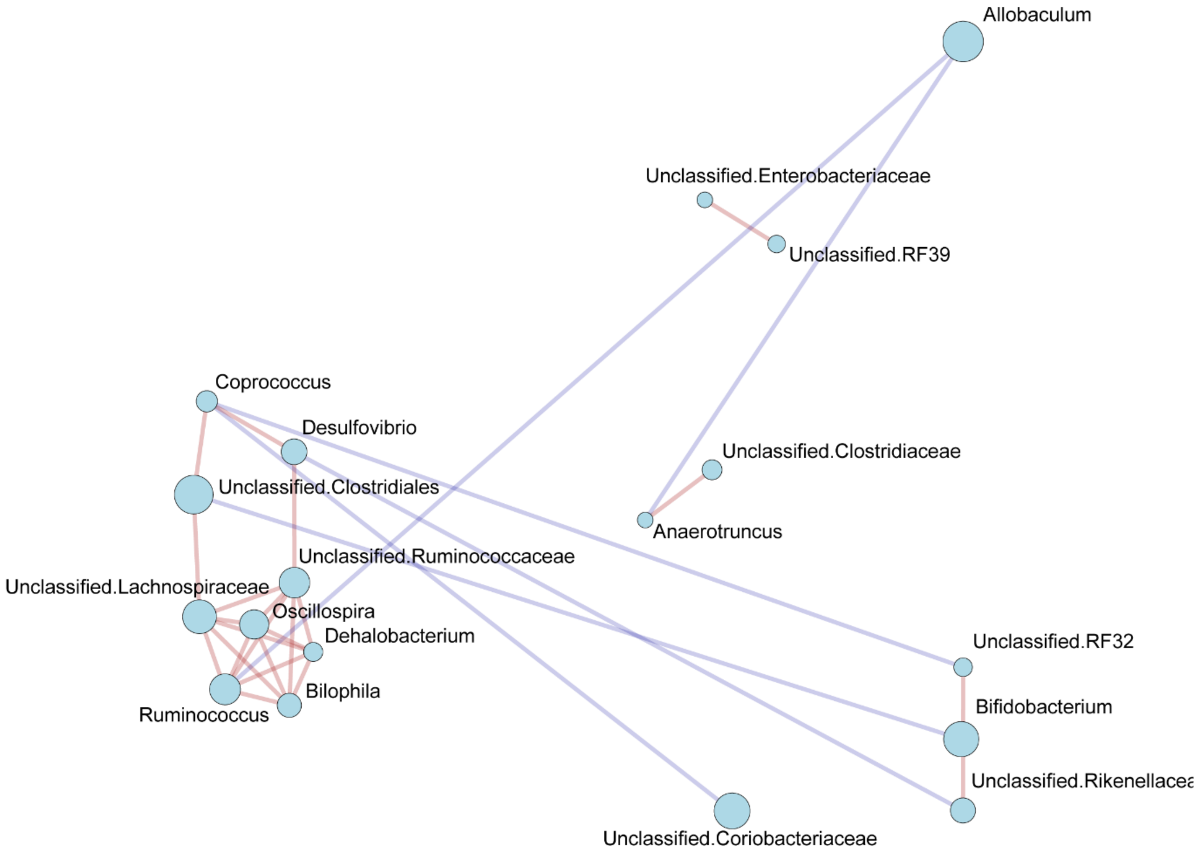
Allobaculum
Unclassified.Enterobacteriaceae
Unclassified.RF39
Coprococcus
Desulfovibrio
Unclassified.Clostridiaceae
Unclassified.Clostridiales
Anaerotruncus
Unclassified.Ruminococcaceae
Unclassified.Lachnospiraceae
Oscillospira
Dehalobacterium
Bilophila
Ruminococcus
Unclassified.RF32
Bifidobacterium
Unclassified.Coriobacteriaceae

I

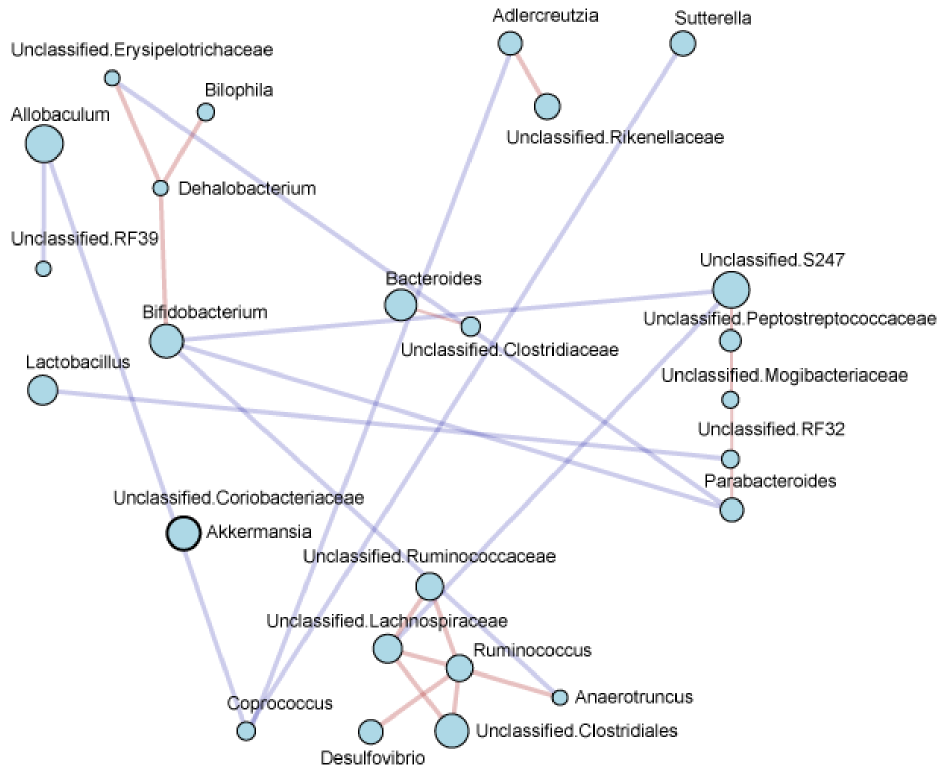
Adlercreutzia
Sutterella
Unclassified.Erysipelotrichaceae
Bilophila
Allobaculum
Unclassified.Rikenellaceae
Dehalobacterium
Unclassified.RF39
Unclassified.S247
Bacteroides
Bifidobacterium
Unclassified.Peptostreptococcaceae
Unclassified.Clostridiaceae
Lactobacillus
Unclassified.Mogibacteriaceae
Unclassified.RF32
Parabacteroides
Unclassified.Coriobacteriaceae
Akkermansia
Unclassified.Ruminococcaceae
Unclassified.Lachnospiraceae
Ruminococcus
Anaerotruncus
Coprococcus
Unclassified.Clostridiales
Desulfovibrio

J

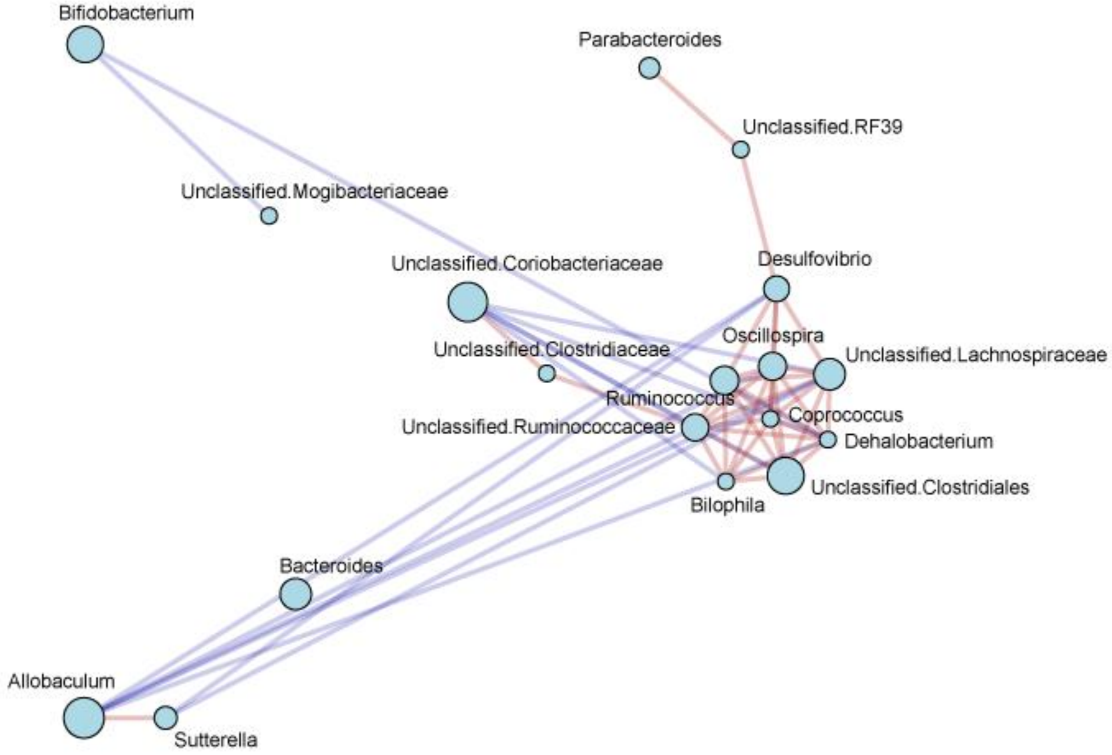
Bifidobacterium
Parabacteroides
Unclassified.RF39
Unclassified.Mogibacteriaceae
Unclassified.Coriobacteriaceae
Desulfovibrio
Oscillospira
Unclassified.Clostridiaceae
Unclassified.Lachnospiraceae
Ruminococcus
Coprococcus
Unclassified.Ruminococcaceae
Dehalobacterium
Unclassified.Clostridiales
Bilophila
Bacteroides
Allobaculum
Sutterella

K

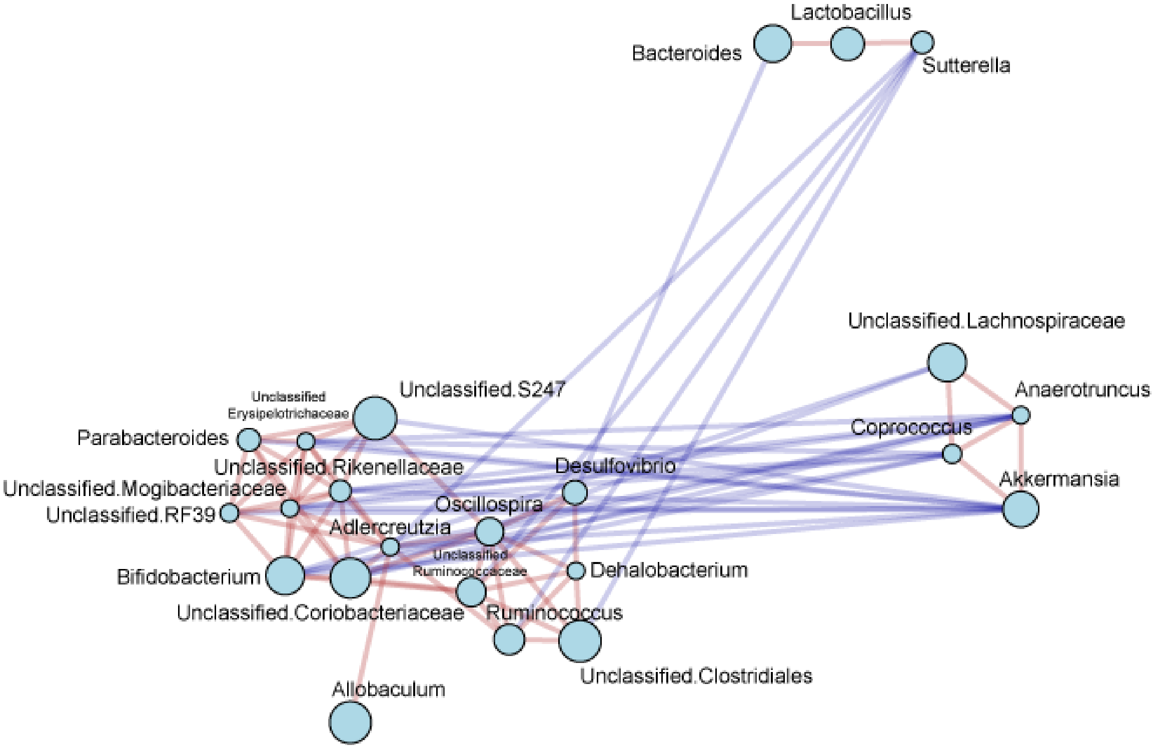

Supplementary Figure 2: Impact of oral administration of TiO_2 on gut microbiota composition

Microbiota composition was determined by 16S rRNA gene sequencing. **(A)** Faith's diversity was determined from colonic microbiota composition from mice treated with 0, 2, 10 or 50 mg TiO_2/kg BW/day in drinking water and **(B-C)** differences in colonic microbiota communities between treatment groups were determined by both Weighted and Unweighted UniFrac PCoA analysis. **(D)** Canonical correspondence analysis ordination of Bray-Curtis dissimilarity of colonic microbiota composition of mice administered 0, 2, 10 or 50 mg TiO_2/kg BW/day in drinking water. CCA was plotted with TiO_2 doses as a continuous variable. Small intestinal microbiota diversity was unchanged as determined by **(E)** Shannon, Inverse Simpson's and Faith's diversity indices as well as richness and evenness. **(F-G)** Weighted and Unweighted UniFrac PCoA analyses on small intestine microbiota revealed no pattern in overall microbial composition. A minimum of n=5 mice per group were analysed. Co-occurrence analysis was performed to examine co-occurring and mutually exclusive bacteria in the microbiome of mice administered **(H)** 0, **(I)** 2, **(J)** 10 or **(K)** 50 mg TiO_2/kg BW/day. This was performed using an ensemble method that combines Pearson's and Spearman's correlation along with Bray-Curtis dissimilarities to determine significant edges (false discovery rate < 0.05). Negative edges are presented as blue lines while positive edges are presented as red lines. Node size represents relative abundance of the genera.

Supplementary Figure 3 related to figure 1

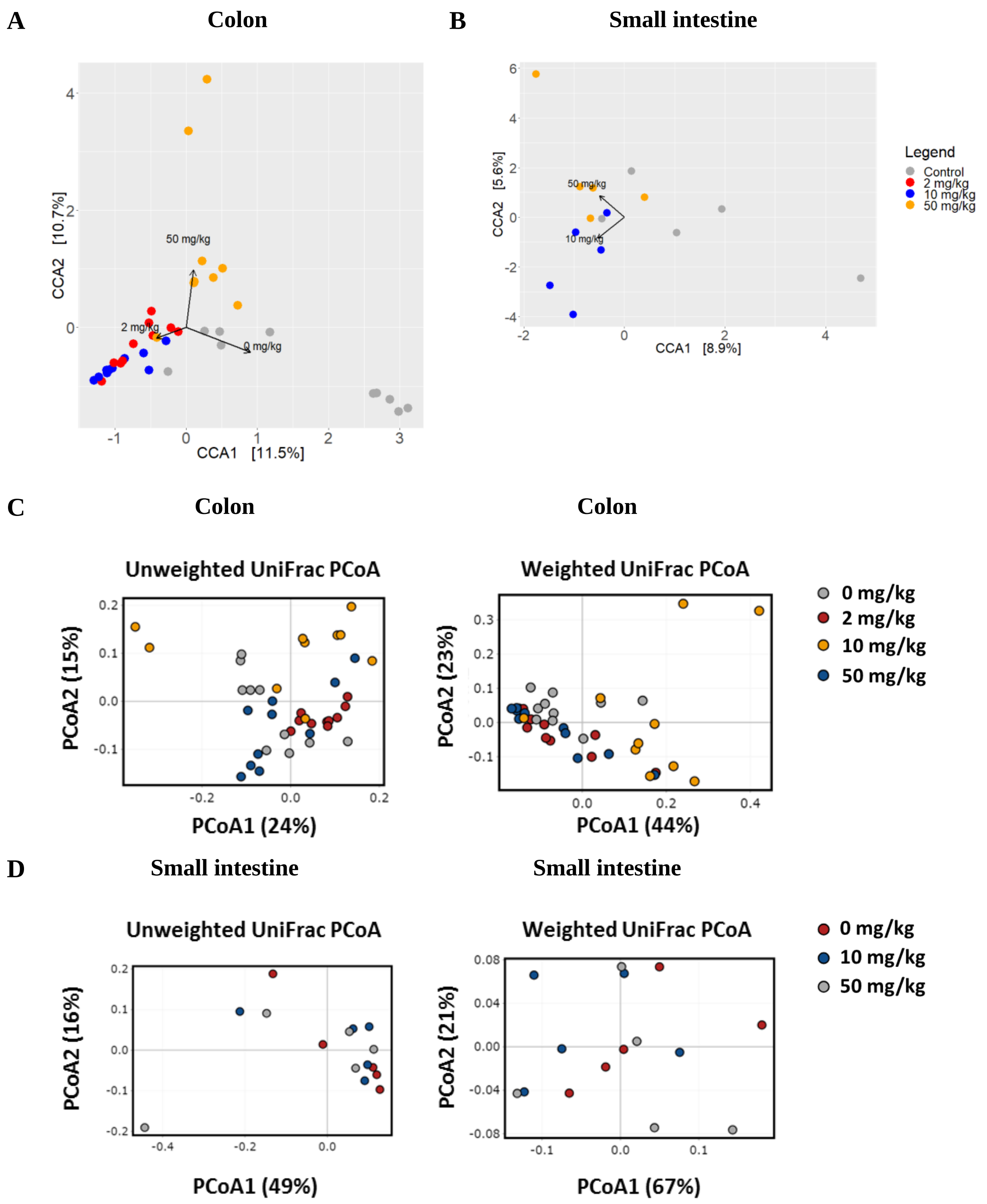

E

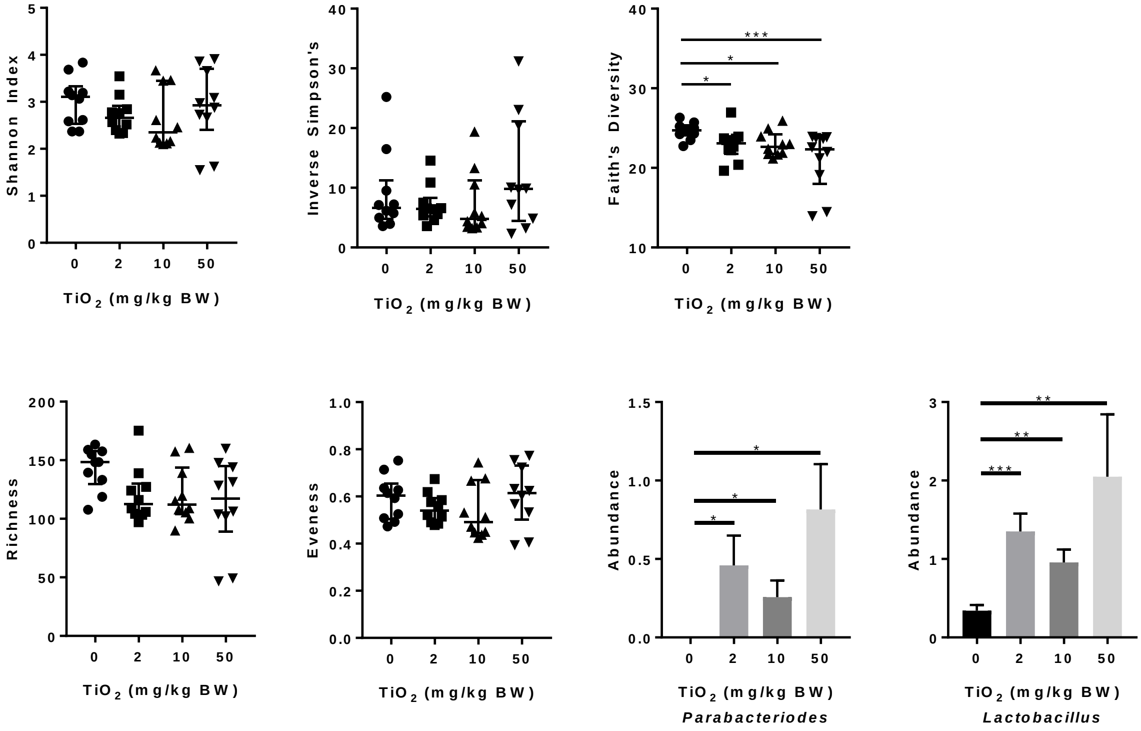

F

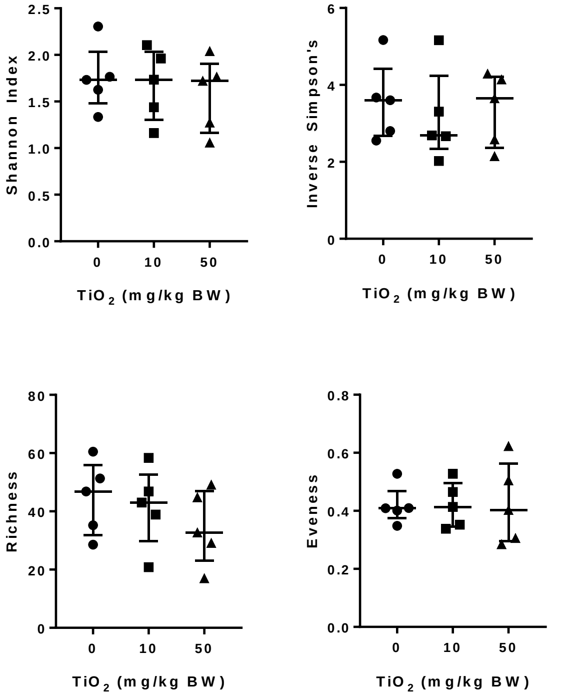

Supplementary Figure 3: Validation of the impact of oral administration of TiO_2 on gut microbiota composition

16S rRNA gene sequencing data was processed through the deblur pipeline. Canonical correspondence analysis ordination of Bray-Curtis dissimilarity of (A) colonic or (B) small intestinal microbiota compositions of mice administered various doses of TiO_2 in drinking water. Microbiota communities between treatment groups in (C) colon and (D) small intestine were determined by Weighted and Unweighted UniFrac PCoA analysis. (E) Diversity of microbiota composition was determined in the colon by Shannon Index, Inverse Simpson's Index, Faith's diversity as well richness and evenness indices. Relative abundance of *Parabacteroides* and *Lactobacillus* was also determined, based on sequences processed through the deblur pipeline, for comparison. (F) Diversity of the small intestine microbiome was determined by Shannon Index, Inverse Simpson's Index, Faith's diversity as well richness and evenness indices. A minimum of n=5 mice per group were analysed. Data are represented as median +/- IQR. *$p < 0.05$, ***$p < 0.005$ as determined by Mann–Whitney U test.

Supplementary Figure 4 related to Figure 3

A

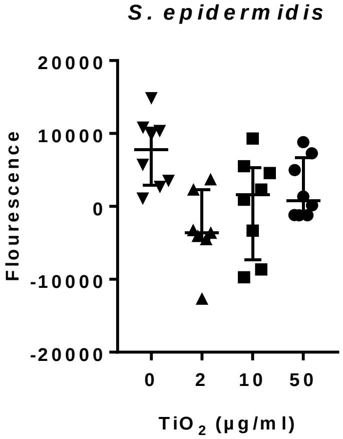

Supplementary Figure 4: TiO_2 does not promote biofilm formation in non-biofilm making bacteria

The ability of *Staphylococcus epidermidis* to form biofilm *in vitro* was assessed in the presence of 0, 2, 10 or 50 µg/ml TiO_2. **(A)** Biofilm formation was assessed by Resazurin viability assay on non-planktonic bacteria after culture. n=8 replicates per condition and 2 independent experiments were performed. Data represented as median +/- IQR.

Supplementary Figure 5 related to Figure 3

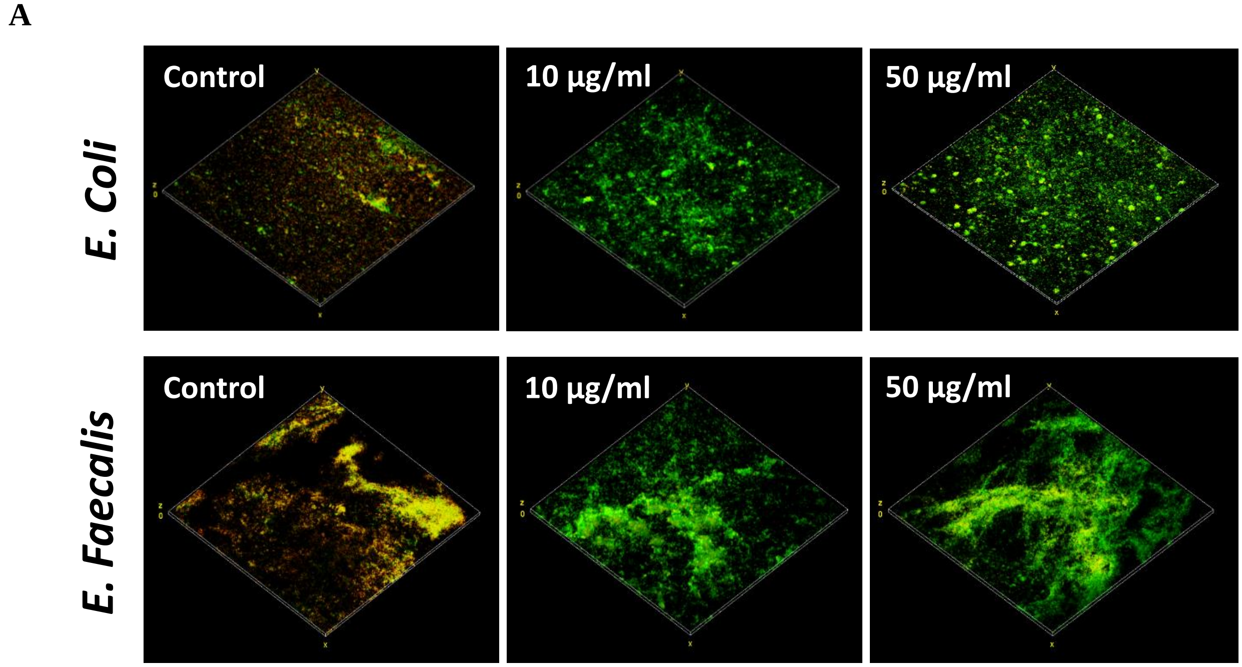

Supplementary Figure 5: TiO_2 triggers biofilm formation by commensal bacteria *in vitro*

(A) Biofilm formation was assessed by confocal microscopy on *E. Coli* or *E. Faecalis* cultured for 7 days with 0 (control), 10 or 50 µg/ml of TiO_2. Biofilm was visualised with the Baclight LIVE/DEAD stain.

Supplementary Figure 6 related to Figure 4

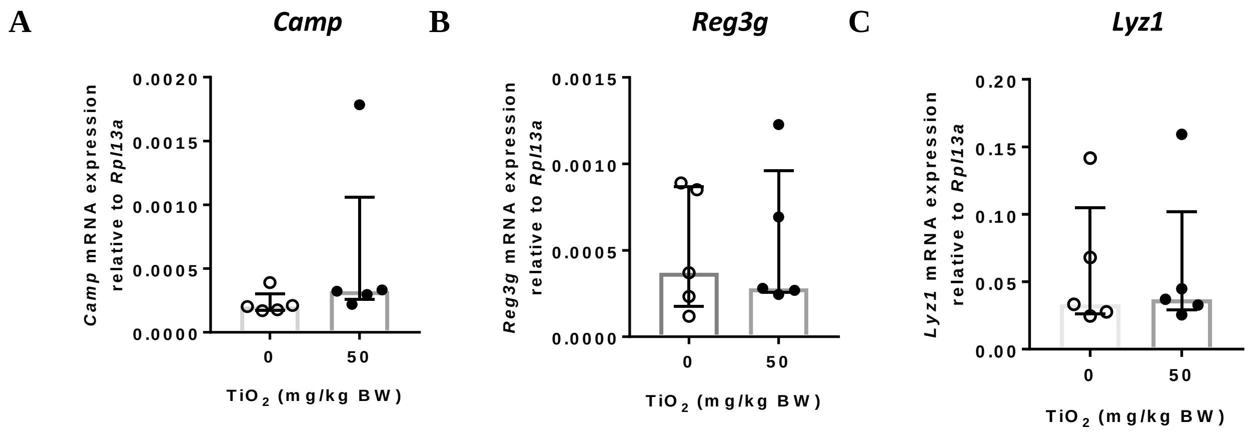

Supplementary Figure 6: TiO_2 affects colonic epithelial gene expression The impact of TiO_2 on colonic epithelial function was determined by comparison of gene expression of **(A)** *Camp* **(B)** *Reg3g* and **(C)** *Lyz2* in colonic tissue of mice administered 0, or 50 mg TiO_2/kg BW/day in drinking water. n=5 mice per group. Data represented as median +/- IQR.

Supplementary Figure 7 related to Figure 5

A

Monocytes

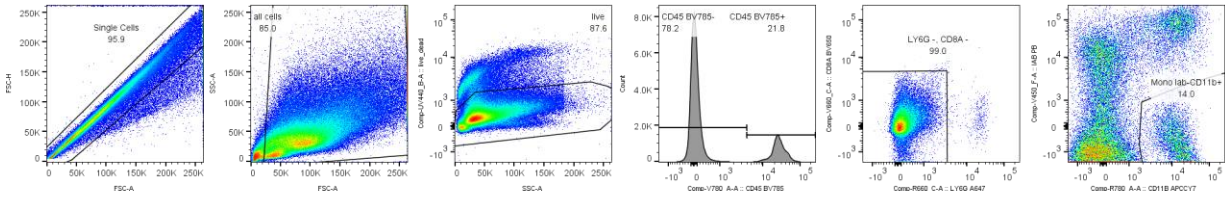

B

Macrophages

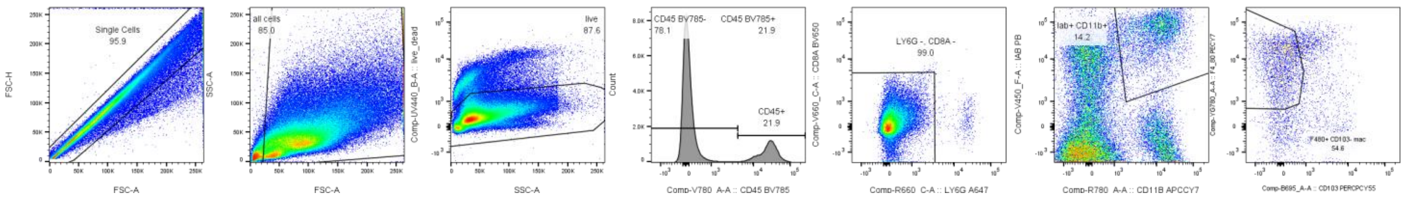

C

Neutrophils

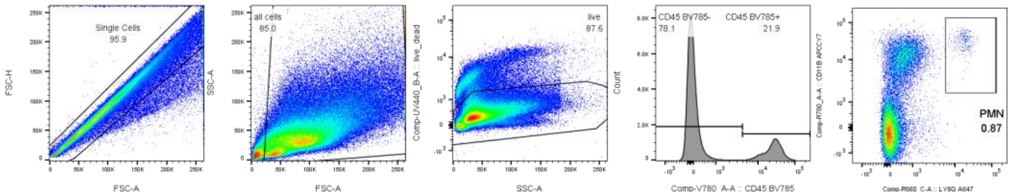

Supplementary Figure 7: Gating strategies of flow cytometry analysis of colonic myeloid populations

Colon tissues were digested and resulting leukocytes analysed by flow cytometry. Representative back gating for **(A)** monocytes, **(B)** macrophages and **(C)** neutrophils are shown.

Supplementary Figure 8 related to Figure 6

A

Th17 T cells

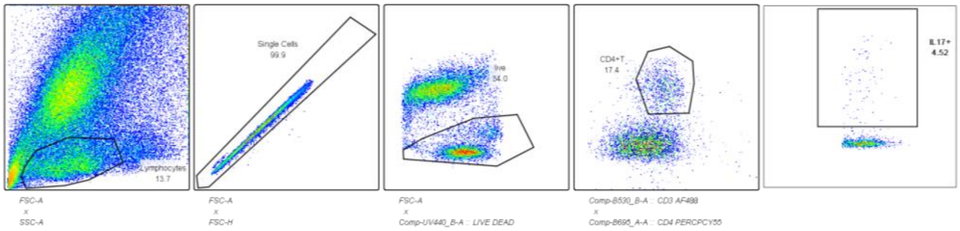

B

Tregs

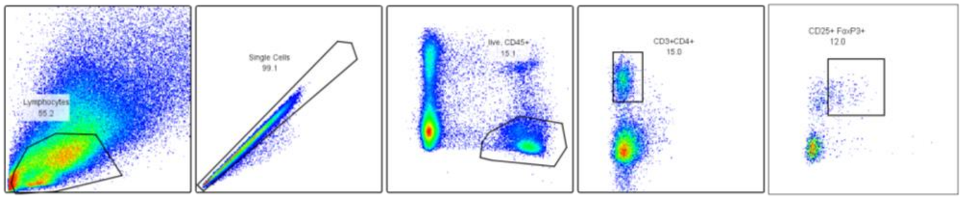

.

C

CD8 T cells

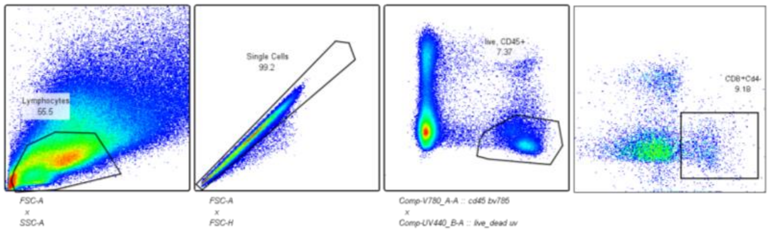

Supplementary Figure 8: Gating strategies of flow cytometry analysis of colonic lymphoid populations

Colon tissues were digested and resulting leukocytes analysed by flow cytometry. Representative back gating for (a) Th17 producing T cells, (b) regulatory T cells and (c) CD8^{+} T cells are shown.
